# Ammonia Sensing via Pseudo Molecular Doping in UV-Activated Ambipolar Silicon Nanowire Transistors

**DOI:** 10.1021/acsami.5c08140

**Published:** 2025-07-24

**Authors:** Vaishali Vardhan, Subhajit Biswas, Leonidas Tsetseris, Sayantan Ghosh, Ahmad Echresh, S. Hellebust, Rene Huebner, Yordan M. Georgiev, Justin D. Holmes

**Affiliations:** † School of Chemistry, 8795 University College Cork, Cork, T12 YN60, Ireland; ‡ Environmental Research Institute, University College Cork, Cork, T23 XE10, Ireland; § Department of Physics, School of Applied Mathematical and Physical Sciences, 68994 National Technical University of Athens, Athens 15780, Greece; ∥ Institute of Ion Beam Physics and Materials Research, 28414 Helmholtz-Zentrum Dresden Rossendorf, 01328, Dresden, Germany; ⊥ Technische Universität Dresden, Dresden, 01069, Germany; # Institute of Electronics at the Bulgarian Academy of Sciences, 1784 Sofia, Bulgaria

**Keywords:** silicon nanowire, junctionless transistor, ambipolar device, molecular doping, density functional theory, ammonia sensing, UV-enhanced sensing

## Abstract

The potential of adsorbed gaseous molecules to create shallow electronic states for thermally excited charge carrier transport and to engineer silicon transistor properties has been largely overlooked compared to traditional substitutional impurities. This paper successfully modifies the electrical properties of ambipolar silicon junctionless nanowire transistors (Si-JNTs) using the reducing properties of ammonia (NH_3_) for selective detection. Physisorption of NH_3_ induces a dual response in both *p*- and *n*-type conduction channels of ambipolar Si-JNTs, significantly altering current and key parameters, including the “on” current (*I*_on_), threshold voltage (*V*_th_), and mobility (μ). NH_3_ interaction increases conduction in the *n*-channel and decreases it in the *p*-channel, acting as an electron donor and hole trap, as supported by Density Functional Theory (DFT) calculations. This provides a pathway for charge transfer and ″pseudo″ molecular doping in ambipolar Si-JNTs. This NH_3_-mediated molecular doping and conduction modulation in Si transistor enabled, for the first time, the electrical detection of gaseous NH_3_ at room temperature across a wide concentration range (200 ppb to 50 ppm), achieving high sensitivity (200 ppb) and precise selectivity under ultraviolet (UV) light. UV illumination dynamically modulates current and reveals distinct sensing features in the *p*- and *n*-channels of the dual-responsive Si-JNTs. The ambipolar Si-JNT sensor exhibits a fast response time of 1.91 min for 0.8 ppm of NH_3_ in the hole conduction channel and a high sensitivity of 80% for 0.8 ppm of NH_3_ in the electron conduction channel. This dual-channel approach optimizes sensor performance by leveraging the most responsive parameters from each channel. Furthermore, the ambipolarity of Si-JNTs broadens the parameter space for developing a multivariate calibration model, enhancing the selectivity of Si-JNT sensors for NH_3_ detection.

## Graphical abstract

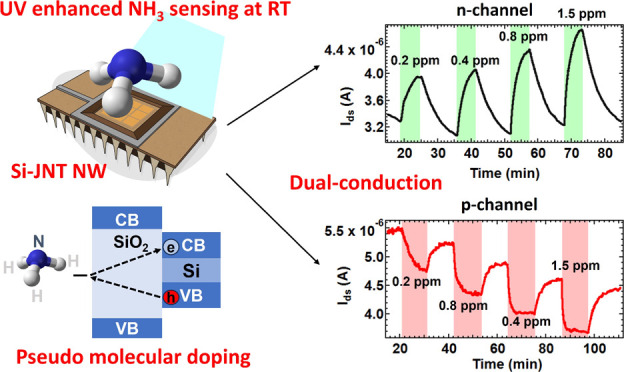

## Introduction

Ammonia (NH_3_), primarily emitted from animal-based agriculture (∼99%), is a major contributor to air pollution in Europe.^1^ Urban vehicle emissions also produce localized high NH_3_ concentrations,^2,3^ which react with acidic compounds such as sulfur dioxide (SO_2_) and nitrous oxides (NO_2_ and NO) to form particulate matter, contributing to transboundary air pollution.^4–7^ At high concentrations, NH_3_ can damage skin, eyes, and respiratory tissues, with prolonged exposure potentially causing severe health issues.^7,8^ NH_3_, a toxic industrial and environmental gas, has exposure limits of 25 ppm for 8 h work periods and 35 ppm for short-term (15 min) exposure.^9^ In farming and medical settings, lower detection thresholds (under 10 ppm) are often preferred to ensure early warnings and adherence to safety standards.^10^

Current solid-state NH_3_ detection sensors, such as metal oxide-based chemresistive sensors, field-effect transistor sensors and electrochemical sensors, while portable, often lack sufficient sensitivity, selectivity, energy efficiency, and real-time detection.^11,12^ Although metal oxide sensors are cost-effective and widely used, they typically require high operating temperatures.^13,14^ Advances in conducting polymer-based NH_3_ sensors have achieved high sensitivity at room temperature, but long-term stability remains an issue.^15^ Optical sensors based on absorption spectroscopy provide accurate real-time detection, but their complexity and cost limit widespread adoption.^16^

Silicon nanowire transistors, renowned for their superior electrostatic control and scalability, have enabled the development of smaller, faster, and more energy-efficient devices in various functional nanosystems, including three-dimensional integrated electronics.^17–19^ Among them, silicon junctionless nanowire transistors (Si-JNTs) stand out due to their unique characteristics, including the absence of gated junctions, a high surface area-to-volume ratio, and the ability to interact chemically with surface analytes to modulate device properties. These attributes make Si-JNTs exceptionally sensitive to changes in electrostatic potential on their channel surfaces.^20–24^ First-principles calculations suggest that the molecular-based *ex-situ* doping, where ammonia (NH_3_) is adsorbed on Si nanowire surfaces, offers an alternative pathway for *n*-type doping by acting as a shallow donor.^25^

Ambipolar transistors, capable of supporting positive (hole) and negative (electron) charge transport^26^ have gained attention in metal oxide semiconductor devices, light-emitting transistors, memory units, neuromorphic computing systems and sensors.^26,27^ Interaction with a reducing agent, like NH_3_, induces contrasting effects in ambipolar devices by increasing electron conductivity while decreasing hole conductivity.^28,29^ This external molecular adsorption offers a dynamic alternative to traditional doping using substitutional impurities, significantly changing transistor electrical properties.^30^ The ability of transistors to dynamically respond to gas molecules provides an opportunity for precise gas detection.

Developing NH_3_ sensors on a scalable, reliable silicon transistor platform offers several advantages over alternative materials like graphene,^31,32^ conductive polymers,^33,34^ carbon nanotubes,^35,36^ and their composite.^37,38^ Si nanowires exhibit high carrier mobility, sensitivity to surface-adsorbed analytes, and compatibility with existing Si semiconductor technology.^39,40^ Si-JNTs, with their simple fabrication and high sensitivity to electrostatic changes, are particularly promising.^21,24^ Ambipolar Si-JNTs, which allow both hole and electron transport, offer dual-channel responses that enhance gas detection sensitivity and selectivity.^26,27,29^ Leveraging multiple electrical parameters in ambipolar Si-JNTs further improves gas discrimination.

NH_3_ sensing on Si platforms, such as mesoporous silicon and silicon nanowires, has been explored due to these materials’ high surface area and reactivity. Most existing sensors, however, require surface modifications or hybrid materials like titania, graphene oxide or polyaniline to achieve high sensitivity and fast response time for ppm to subppm of NH_3_ concentrations^41^ (see Table S1 in Supporting Information). For example, Song et al.^42^ used bottom-up grown Si nanowire transistors to achieve 100 ppb NH_3_ sensitivity with 7% responsivity. However, challenges remain for achieving performance at subppm concentrations in simpler systems. While bare Si nanowire field effect transistor sensors show moderate response times (∼86 s for 5 ppm of NH_3_) and high selectivity, recovery remains a challenge.^43^ Functionalized variants like polyaniline-Si nanowires substantially increase response speed (32 s for 1 ppm of NH_3_) and decrease the detection limit to 1 ppb, though they exhibit diminished operational efficacy and stability.^44^ Hybrid methods, such as reduced graphene oxide/ZnO on Si nanowires, achieve remarkably fast response times (3 s for 0.01 ppm of NH_3_) and strong signal strengths; however, they struggle with performance issues due to humidity variations.^45^ Addressing long recovery times and stability issues remains crucial for real-world applications. Transistor-based architectures with gate-induced signal amplification and UV-driven carrier generation could address these limitations. Recent advances have demonstrated that UV light significantly enhances NH_3_ sensing performance on silicon platforms^46^ (see Table S2 in Supporting Information). Highly engineered materials, such as Se- or S-hyperdoped nanowires, have achieved ppm-level NH_3_ sensing under UV light. However, using UV light to enhance subppm of NH_3_ detection on simple Si platforms without surface modifications or hybrid materials remains largely unexplored. In particular, the dual interaction of NH_3_ and UV light with ambipolar Si-JNTs offers a promising approach for low-power, highly sensitive, and fast-recovery NH_3_ sensors.

This paper discusses how NH_3_ acts as a pseudodopant for Si-JNTs, altering electrical properties in both *p*- and *n*-channels. Density Functional Theory (DFT) calculations confirm the NH_3_-induced changes in conduction and charge transfer. These interactions dynamically control multiple electrical parameters in ambipolar Si-JNTs, enabling the sensitive detection of NH_3_ under the influence of UV light (λ = 254 nm). Additionally, we investigate the effect of UV light (λ = 254 nm) on key sensor parameters, such as responsivity, response time, and recovery time, in both the conduction channels of the ambipolar transistor, achieving efficient NH_3_ detection at subppm concentrations (250 ppb -2 ppm). This research represents the first demonstration of UV-driven gas sensing in Si-JNT devices.

## Experimental Section

### Fabrication of Si-JNTs

Si-JNT devices were fabricated on ultrathin SOI substrates with 20 nm wide, 6 μm high nanowire channels. Phosphorus doping was performed via chain implantation, followed by flash lamp annealing (FLA)^47^ for activation and defect repair. Nanowires were patterned using electron beam lithography (EBL) and reactive ion etching (RIE).^48^ Nickel–gold contacts were deposited using UV lithography, metal evaporation, and lift-off, followed by rapid thermal annealing (RTA) at 450 °C to form nickel silicide. Hall-effect measurements confirmed an *n*-type carrier concentration of ∼6 × 10^17^ cm^–3^. Postmetal RTA conditions influenced device ambipolarity. A uniformly grown thin thermal oxide layer was grown via an oxidation process conducted at a temperature of 900 °C under an O_2_ atmosphere for several seconds. A comprehensive account of the device fabrication and characterization process can be found in previous papers.^49,50^

### Characterization of Si-JNTs

The morphology and structure of Si-JNTs were analyzed using a FEI Quanta 650 scanning electron microscope (SEM) and a Titan 80 operating at 300 kV. Cross-sectional TEM specimens of the Si-JNT devices were prepared *in situ* using a Helios 5 CX system (Thermo Fisher) for lift-out. A protective carbon layer was deposited before FIB milling, and final thinning to electron transparency was performed using low-energy (5 keV) Ga ions to minimize sidewall damage. Energy Dispersive X-ray (EDX) analysis was conducted to determine the elemental composition of the Si-JNT devices in conjunction with FEI Titan 80. The electrical characterization of the Si-JNTs was performed using an analysis setup comprising two Keithley 2450 source meters connected to a Nextron (South Korea) microprobe station with a 100 cm^3^ volume. All electrical measurements, including *I*-*V* characteristics, were conducted using Keithley Kickstart software, version 2.2.1. The machine learning computations and analyses were performed using MATLAB R2022a.

### Characterization of Si-JNT NH_3_ Sensors Under Ambient and UV Conditions

Si-JNTs were exposed to various NH_3_ environments with mixing ratios ranging from 200 ppb to 50 ppm, and their responses were compared. The interaction between NH_3_ and Si-JNTs was studied using a semicustomized gastight microprobe station (Nextron). A flow of zero air (ZA), a mixture of nitrogen and oxygen and free of trace gases, particles, and humidity, was introduced at a rate of 5 standard liters per min (SLPM) for 20 min, followed by NH_3_ exposure for 10–15 min, at specific mixing ratios depending on the experiment. All measurements were taken at room temperature, defined as 20–23 °C in this study. To assess a sensor’s performance, the transfer characteristics of Si-JNTs were recorded under ZA and at each NH_3_ exposure level. Key transistor parameters, including transconductance (*g*_m_), threshold voltage (*V*_th_), field-effect mobility (μ), and on-current (*I*_on_), were evaluated, along with the impact of NH_3_ concentration on these parameters. *V*_th_ was extracted using the transconductance derivative method at a low drain bias (*V*_ds_ = 1 V).^50^ Typical field-effect mobility was calculated from the linear region of the transfer characteristic curves, using the transconductance value, capacitance, and other nanowire geometrical parameters.^51,52^ Additional parameters, such as response time, responsivity, recovery time, and device stability, were extracted from the time-dependent current evolution curves at fixed gate and drain voltages. For gas sensing experiments under UV light, a 254 nm light source was positioned above the probe station and covered with a black cloth to maximize light concentration on the sample. The time-dependent current was recorded to evaluate the sensor parameters while maintaining a fixed drain-to-source voltage (*V*_*ds*_) of 1 V. The gate-to-source voltage (*V*_*gs*_) was set to −40 V for the *p*-channel and 40 V for the *n*-channel. A baseline measurement was first recorded in the presence of ZA. Then, 1 ppm of NH_3_ gas was introduced to the Si-JNT device for 10 min for the *p*-channel and 12 min for the *n*-channel. Throughout the experiment, UV light was continuously present. Before each experiment, the samples were evacuated under vacuum for 30 min. For the *p*-channel, the drain-to-source voltage (*V*_ds_) and gate-to-source voltage (*V*_gs_) were set to 1 V and −40 V, respectively. For the *n*-channel, *V*_ds_ and *V*_gs_ were maintained at 1 and 40 V, respectively. The source-to-drain current (*I*_ds_) was recorded during exposure to ZA and NH_3_ at various concentrations. All sensing measurements were carried out with the UV light source active throughout the experiment.

### Density Functional Theory (DFT) Calculations

Quantum-mechanical DFT calculations were undertaken to elucidate the atomic-scale interactions between NH_3_ molecules and the oxidized surfaces of the Si nanowires (in the Si-JNTs). Calculations were carried out using the DFT code VASP,^53^ employing a 500 eV energy cutoff, projector augmented waves (PAWs),^54^ and the generalized gradient approximation (GGA) Perdew–Burke–Ernzerhof^55^ exchange-correlation (xc) functional. Nonbonding van der Waals interactions were accounted for within the DFT-D3 scheme.^56^ Structural representations were generated using the software VESTA.^57^

## Results and Discussion

Si-JNTs with a back-gate configuration and an array of 20 nanowires were used for NH_3_-mediated pseudomolecular doping and subsequent detection (see the Experimental Section in the Supporting Information for details on device fabrication and characterization). The cross-sectional schematic of a Si-JNT device and a top-view SEM image of the 20 nanowire array are shown in Figures 1(a) and 1(b), respectively. All Si-JNT devices feature a channel length of 6 μm. The nanowires have a height of 20 nm and consist of a single crystalline Si core surrounded by a 1–2 nm thick native oxide layer, as confirmed by the cross-sectional high-resolution TEM image shown in Figure 1(c). Analysis based on EDX analysis was used to determine the elemental composition of the Si-JNT devices, as shown in Figure 1(d). Unlike most gas-phase sensors based on silicon transistors, the Si-JNT sensor for NH_3_ detection does not contain any additional elements, as shown in the maps in Figure 1(d). This Si–O surface allows direct physisorption of NH_3_, enabling charge transfer through tunnelling across the oxide barrier on nanowire surfaces.^47–49^ The typical drain current (*I*_ds_) vs. gate voltage (*V*_gs_) characteristics of a Si-JNT device with a 1–2 nm native oxide layer are shown in Figure 1(e). These transfer characteristics were recorded at a constant drain-to-source voltage (*V*_ds_) of 1 V, using a butterfly sweep of the gate-to-source voltage (*V*_gs_) from −30 to 30 V. The unpassivated ambipolar devices exhibit highly symmetric ambipolarity, with similar current levels in both the *p*- and *n*-conduction channels. Both channels ″on″ current (*I*_on_) fall within the 2–3 μA range. The field effect mobility, calculated from the transfer characteristic curve (Figure 1(d)), was 307 cm^2^ V^–1^ s^–1^ for holes and 333 cm^2^ V^–1^ s^–1^ for electrons, indicating balanced electron and hole transport capabilities. The threshold voltages (*V*_th_) for the *p*- and *n*-channels were −24.4 and 22.3 V, respectively and were determined using the transconductance derivative method at a low drain voltage of 1 V.

**Figure 1:**
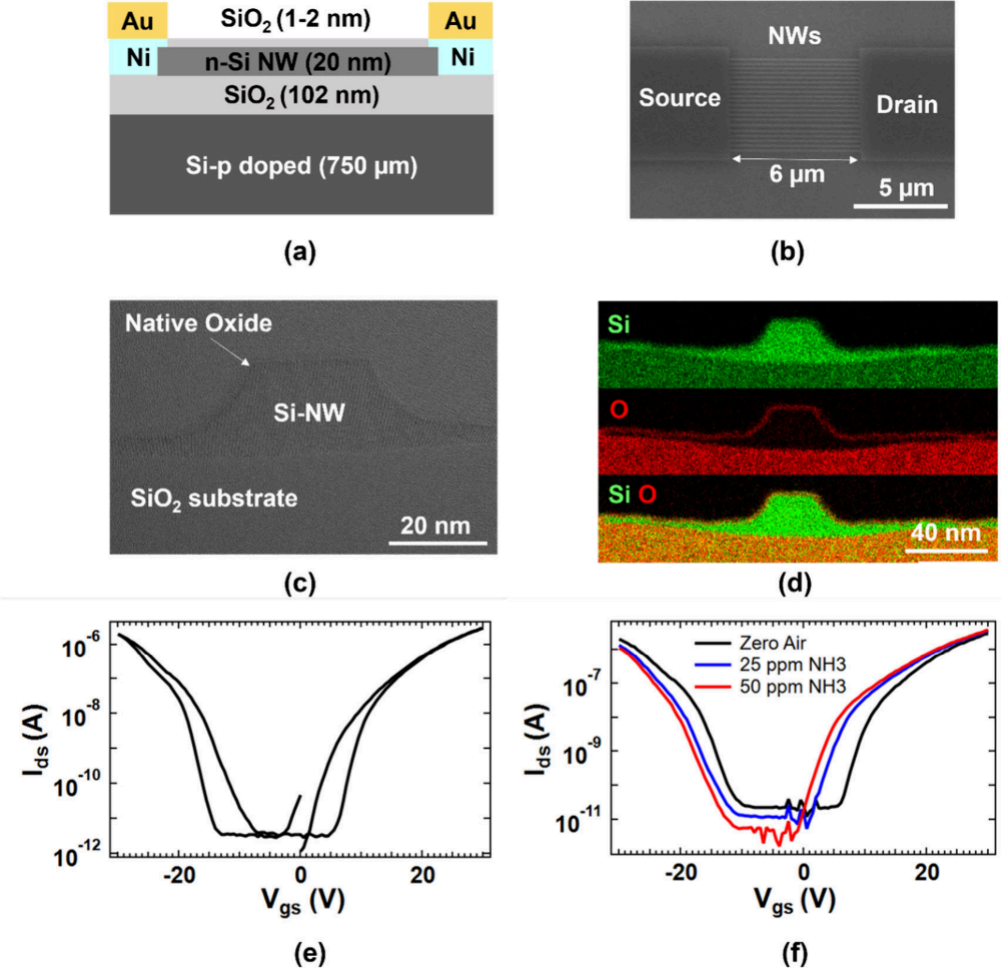
(a) Schematic representation of the Si-JNT device. (b) Top-view SEM image of a Si-JNT nanowire array device. (c) Cross-sectional high-resolution TEM image of a Si-JNT device showing a native oxide layer (1–2 nm). (d) Element distribution analysis of a Si-JNT with native oxide, with Si displayed in green and O in red. (e) Transfer characteristics of an unpassivated ambipolar Si-JNT with a native oxide layer, recorded at a constant *V*_sd_ of 1 V. (f) Evolution of transfer curves under NH_3_ exposure (0, 25 ppm, and 50 ppm) at *V*_sd_ of 1 V and *V*_gs_ from −30 to 30 V.

In the ambipolar Si-JNT devices, hole conduction occurs under reverse gate voltages, while electron conduction is observed under forward gate voltages. Rapid thermal annealing (RTA) of Si-JNT devices forms Ni silicide, resulting in Schottky contact behavior (see Figure S1 in Supporting Information), where *I*_ds_ depends nonlinearly on *V*_ds_. The output characteristics of unpassivated Si-JNT devices were recorded at different gate voltages (−40 to 40 V at an interval of 10 V) while sweeping *V*_ds_ from −1 to 1 V (Figure S1). Due to the lower Schottky barrier height for holes, *p*-type conductance emerges during the back gate sweep, making the device behave like a reconfigurable field-effect transistor (RFET) rather than a typical unipolar Si-JNT. The lower Schottky barrier height for holes leads to *p*-type conduction during back-gate voltage sweeps, producing ambipolar behavior.^58,59^ Additionally, recombination during the *n*-doping process of the initial *p*-type SOI substrate reduces the *n*-dopant concentration while retaining hole carriers in the nanowire channels, further supporting the ambipolarity behavior of Si-JNTs. For more detailed information on the origin of the ambipolarity in Si-JNTs, please refer to our previous publication.^47^

### Effect of NH_3_ on Si-JNT Conduction and Molecular Doping

Electrical tests were conducted in a gastight microprobe station to investigate the interaction of NH_3_ with Si-JNTs, and the devices were exposed to NH_3_ concentrations of 25 to 50 ppm. Ambipolar Si-JNTs with native oxide were tested to evaluate the potential for modifying electrical conduction in both *p*- and *n*-channels. Upon exposure to NH_3_, a noticeable shift in the transfer curves was observed, as shown in Figure 1(f). The *I*–*V* characteristics retained ambipolar behavior, but the drain current in both *p*- and *n*-conduction channels exhibited significant shifts in response to NH_3_ at varying concentrations. A ″dual reaction″ was identified during the interaction of NH_3_ with the Si-JNT. Specifically, the *n*-conduction channel exhibited an increase in drain current with increasing NH_3_ concentration (from 25 to 50 ppm), while the *p*-conduction channel showed a decrease. For the *p*-conduction channel, the ″on″ current at −30 V decreased from 2.06 μA to 1.34 μA and 1.28 μA as the NH_3_ concentration increased to 25 and 50 ppm, respectively. In contrast, the *n*-type ″on″ current at +30 V increased from 2.9 μA to 3.51 μA and 3.56 μA under the same NH_3_ exposure (Figure 2(a)). The hole channel demonstrated a relative decrease in ″on″ current of 30% and 37.9%, while the electron channel exhibited increases of 21.0% and 22.8% for 25 and 50 ppm of NH_3_ exposure, respectively. This fluctuation in *p*- and *n*-channel currents under NH_3_ exposure highlights a mechanism for dynamically controlling the balance and degree of ambipolarity in Si-JNTs. Additionally, NH_3_ exposure caused notable changes in the threshold voltage (*V*_th_) and field-effect mobility. For the hole conduction channel, *V*_th_ shifted from −23.5 V to −24.2 V and −24.3 V with 25 and 50 ppm of NH_3_ exposure, respectively, while the electron conduction channel’s *V*_th_ decreased from 20.9 to 20.4 and 20.3 V (see Figure 2(b)).

**Figure 2:**
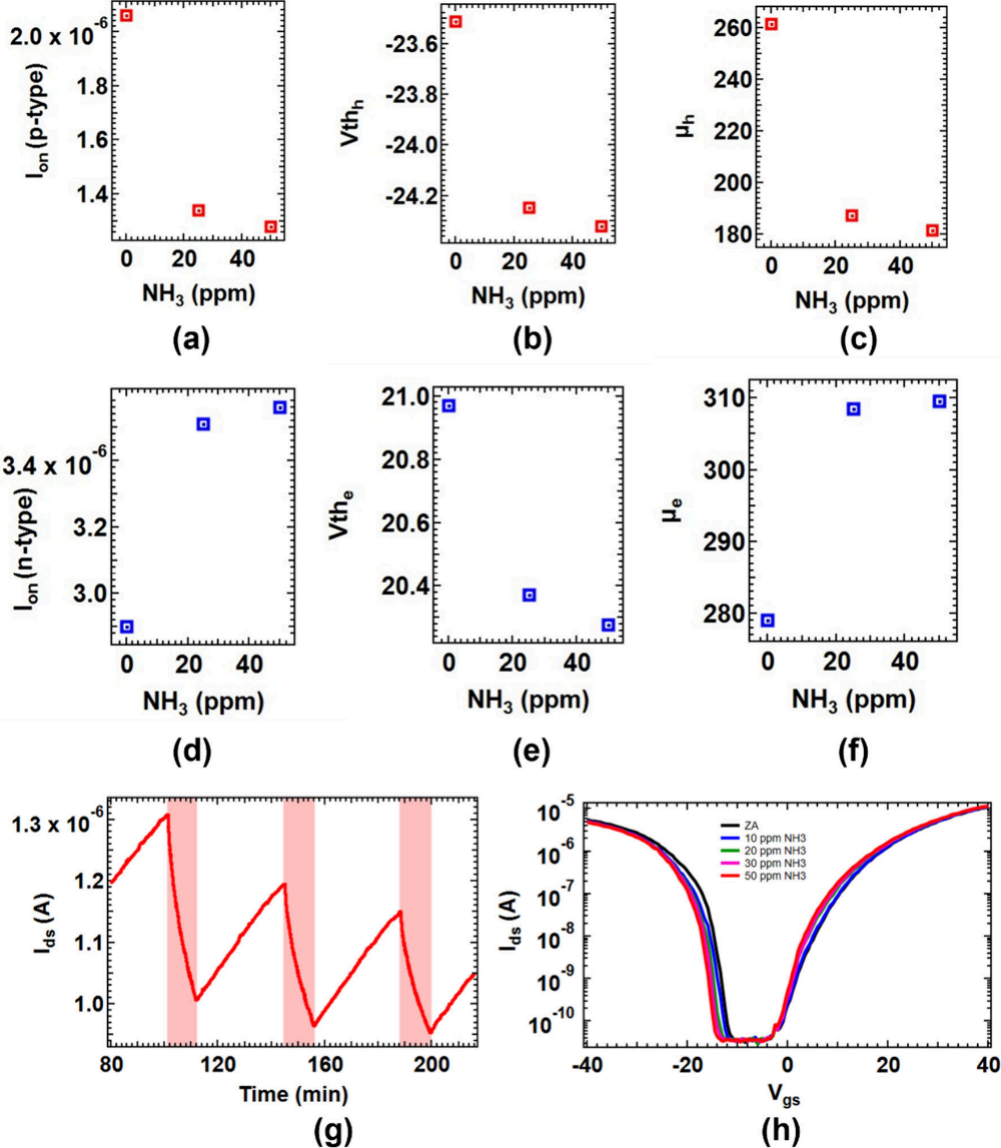
(a) Comparison of “on” current (*I*_on_), (b) mobility (μ),and (c) threshold voltage (*V*_th_) for the *p*-channel of a native oxide Si-JNT upon exposure to 25 and 50 ppm of NH_3_. (d), (e) and (f) show the respective parameters for the *n*-channel under these conditions. (g) Repeatability test for NH_3_ exposure at 25 ppm (indicated by pink bars for 10 min on the *p*-channel of the Si-JNT at *V*_gs_ = −40 V. (h) Change in transfer characteristics of the Si-JNT device upon exposure to zero air and different mixing ratios of NH_3_ (10, 20, 30, and 50 ppm) at *V*_ds_ = 1 V.

NH_3_ also influenced field-effect mobility. In the hole channel, mobility decreased from 261.7 cm^2^ V^–1^ s^–1^ to 187.2 cm^2^ V^–1^ s^–1^ and 181.4 cm^2^ V^–1^ s^–1^ with 25 and 50 ppm of NH_3_ exposure, respectively. Conversely, electron mobility increased from 279.0 cm^2^ V^–1^ s^–1^ to 308.5 cm^2^ V^–1^ s^–1^ and 309.6 cm^2^ V^–1^ s^–1^ under the same conditions (Figure 2(c)). These findings demonstrate that when NH_3_ molecules are adsorbed on the SiO_2_-terminated facets of Si-JNTs, they can selectively modulate the electrical properties of both *p*- and *n*-channels. This enables external tuning of device characteristics, effectively facilitating ″ex-situ″ adjustment and expanding the functional versatility of ambipolar Si-JNTs. Si-JNTs with a native oxide are much more likely to exhibit hysteresis because of the high density of trap states in the native oxide, as shown in Figure 1(e). Notably, when NH_3_, a reducing analyte, is present, the transfer characteristics of Si-JNT devices display a slight but consistent decrease in hysteresis (see Figure S2 in the Supporting Information). NH_3_ donates electrons to the semiconductor channel, which can partially neutralize existing surface or interface trap states. This charge compensation may narrow the memory window between forward and reverse gate sweeps, thus reducing hysteresis. Conversely, exposure to the oxidizing gas NO_2_ results in adsorption on the Si-JNT surface and formation of electron-trapping states during both forward and reverse voltage sweeps, leading to increased hysteresis in unpassivated devices.^49^ These observations emphasize the significant impact of the analyte’s redox nature on the hysteresis behavior of ambipolar Si-JNT sensors.

Dynamic modulation of the conduction in the ambipolar Si-JNT device was recorded for the slightly more responsive *p*-conduction channel. Upon exposure to NH_3_, holes are captured via gas adsorption, potentially causing a shift in the energy bands of the device. This shift results in significant changes to transistor operation. The modulation was measured at fixed *V*_gs_ of −40 V and *V*_ds_ of 1 V, as shown in Figure 2(g). Multiple 10 min pulses of 25 ppm of NH_3_ were introduced to the device, with ZA (at 5 SLPM) for 30 min between each pulse to allow for device recovery. As expected, the current in the *p*-channel decreased with the introduction of NH_3_. The relative conduction change, or responsivity (*R*), was calculated using eq 1, where *R* is responsivity, *I*_on_NH3__ is on current for NH_3_ and *I*_on_ZA__ is on current in the presence of ZA.

$$R=\left(\frac{\left(I_{{on}_{NH3}}-I_{{on}_{ZA}}\right)}{I_{{on}_{NH3}}}\times 100\%\right)$$ 1

Each NH_3_ pulse resulted in responsivities of 23.1, 19.3, and 17.3%, respectively, demonstrating the repeatability of the charge transfer process between NH_3_ molecules and Si-JNTs. These findings indicate that conduction in ambipolar Si-JNTs can be reversibly modulated through the adsorption and desorption of NH_3_ gas molecules at concentrations of 25 ppm. However, the sensor signal does not stabilize during NH_3_ exposure, making it unsuitable to determine response and recovery times in this context accurately. Instead, using the differential current approach is more effective (see Figure S3 in the Supporting Information). This method tracks the rate of signal change, providing better insight into gas interactions, especially for sensors like ambipolar Si-JNTs, where the current continues to increase as the sensor is exposed to gas. This highlights the device’s potential for reliable and repeatable molecular doping with NH_3_. Upon exposure to 50 ppm of NH_3_ in ZA, the hole concentration decreased from 7.5 × 10^18^ to 6.4 × 10^18^ cm^–3^, while electron concentration increased from 1.3 × 10^19^ to 1.4 × 10^19^ cm^–3^. The carrier concentration was calculated using eq 2 from output characteristics (see Figure S4 in the Supporting Information).

2 $$n=\frac{I_{\mathrm{ds}}L}{q\mu V_{\mathrm{ds}}A}$$

where *n* is the carrier concentration, *I*_ds_ is the drain-to-source current, *L* is the distance between source and drain, and *q* is the fundamental charge constant, μ is the mobility, *V*_ds_ is the source to drain voltage, and *A* is the surface area of the nanowire.

### DFT Studies on NH_3_-Induced Changes in Ambipolar Si-JNT Characteristics

DFT calculations were performed to probe the interactions between NH_3_ molecules and the SiO_2_-terminated facets of Si-JNTs. Two potential mechanisms were identified through which NH_3_-related species can alter the concentration of charge carriers (electrons or holes) in the underlying Si active layer. In the first mechanism (Path 1), NH_3_ molecules remain physiosorbed on a defect-free SiO_2_ surface (as shown in Figures 3(a) and 3(b)), introducing filled states within the SiO_2_ energy band gap. The latter are depicted with arrows in the pertinent electronic Density of States (DOS) plots of Figure 3(c), and their energies have a slight dependence on the details of the physiosorbed configuration. These gap states can trap holes from an underlying Si channel, provided the band alignment is suitable, and the oxide layer is sufficiently thin to permit effective tunnelling. It should be noted that filled gap states also occur when an NH_3_ molecule physisorbs near a surface oxygen vacancy, a pair of surface hydroxyl groups, or an adsorbed H_2_O molecule. The related DOS plots and configurations are illustrated in Figure S5 of the Supporting Information.

**Figure 3:**
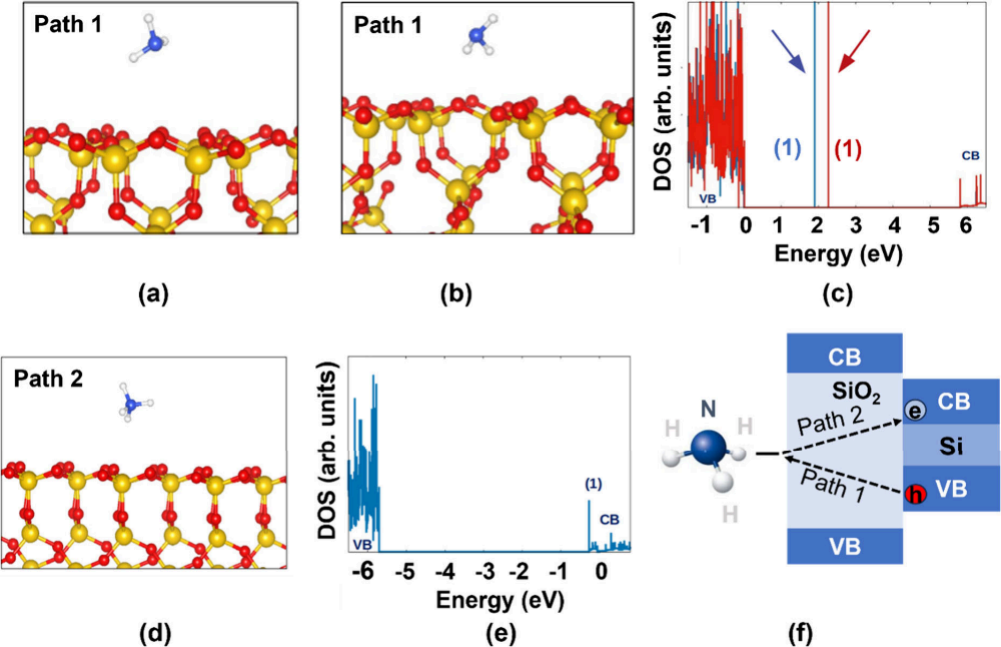
(a–b) Physisorption of NH_3_ molecules on the SiO_2_ surface (Si: yellow, O: red, N: blue, H: white spheres). (c) DOS (arbitrary units) for the configurations in (a) [red line] and (b) [blue line], with the energy zero set at the SiO_2_ valence band maximum (VB = valence band; CB = conduction band). Arrows highlight NH_3_-related states within the SiO_2_ bandgap. (d) Formation of an NH_4_^+^ species resulting from the reaction of NH_3_ with a surface H adatom on SiO_2_. (e) DOS for the configuration in (d), with the energy zero at the highest occupied state, is now positioned within the SiO_2_ CB, indicating electron doping. (f) The Schematic of Paths 1 and 2 shows electron and hole exchange via tunnelling across the SiO_2_ bandgap.

We also found that it is energetically favorable (by 1.24 eV) for an NH_3_ molecule to abstract a hydrogen atom from an OH group on the SiO_2_ surface, resulting in the formation of an NH_4_^+^ species (Path 2, Figure 3(d)). The DOS plot in Figure 3(e) shows that the NH_4_^+^ group creates a filled state at the bottom of the SiO_2_ conduction band. A schematic illustrating charge transfer for both pathways is shown in Figure 3(f). These exothermic reactions facilitate the transfer of electrons to the oxide layer and then to the Si channel’s conduction band via tunnelling. The oxide must be sufficiently thin to allow effective tunnelling. This process is activated when energy states are aligned correctly and is more pronounced with thinner SiO_2_ layers. Holes tunnel through the oxide and become trapped by the physisorbed NH_3_ molecules on its surface. Different oxide thicknesses lead to varying barrier heights for charge tunnelling during NH_3_ adsorption. As a result, the response remains low or negligible (∼ 1.5% at 25 ppm) for Si-JNT with a 10 nm thick oxide, compared to 1–2 nm native oxide layers that exhibit a 22% response at the same NH_3_ concentration.

### UV-Enhanced Ammonia Sensing in Si-JNTs

Ambipolar Si-JNTs exhibit modulation in their electrical characteristics when exposed to ppm concentrations of NH_3_ (Figure 2(h)). The continuous increase in current observed in Si-JNTs under NH_3_ exposure (Figure 2(h)), combined with their highly doped (phosphorus) transistor architecture, narrow channel (∼20 nm), and high surface area, suggests strong potential for enhanced photo response under UV light exposure.^20^ Building on previous studies of Si nanowire transistors’ UV-induced photo response,^60^ high-energy light exposure may enhance NH_3_ sensing across a wide range of concentrations, from high (10–50 ppm) to low (0.2–1.5 ppm).

To confirm this effect, a 254 nm UV light source (4 W) was used in conjunction with NH_3_ exposure to evaluate the response, response time, and recovery time of the ambipolar Si-JNT sensor. A UV wavelength of 254 nm is particularly effective as it provides the necessary driving influence for NH_3_ sensing, with ammonia photodissociation at wavelengths between 100–200 nm.^61^ Several tests were conducted to evaluate repeatability, responsivity, response time, saturation, and recovery of ambipolar Si-JNT sensors in both the *p*- and *n*-conduction channels under UV light exposure. These tests spanned the NH_3_ high concentration regime, ranging from 10 to 50 ppm. As expected, NH_3_ exposure resulted in an increase in current for the *n*-channel and a decrease for the *p*-channel of the Si-JNTs. Notably, the Si-JNTs exhibited a significantly enhanced response to NH_3_ under UV light compared to conditions without illumination in both conduction channels (Figure 4).

**Figure 4:**
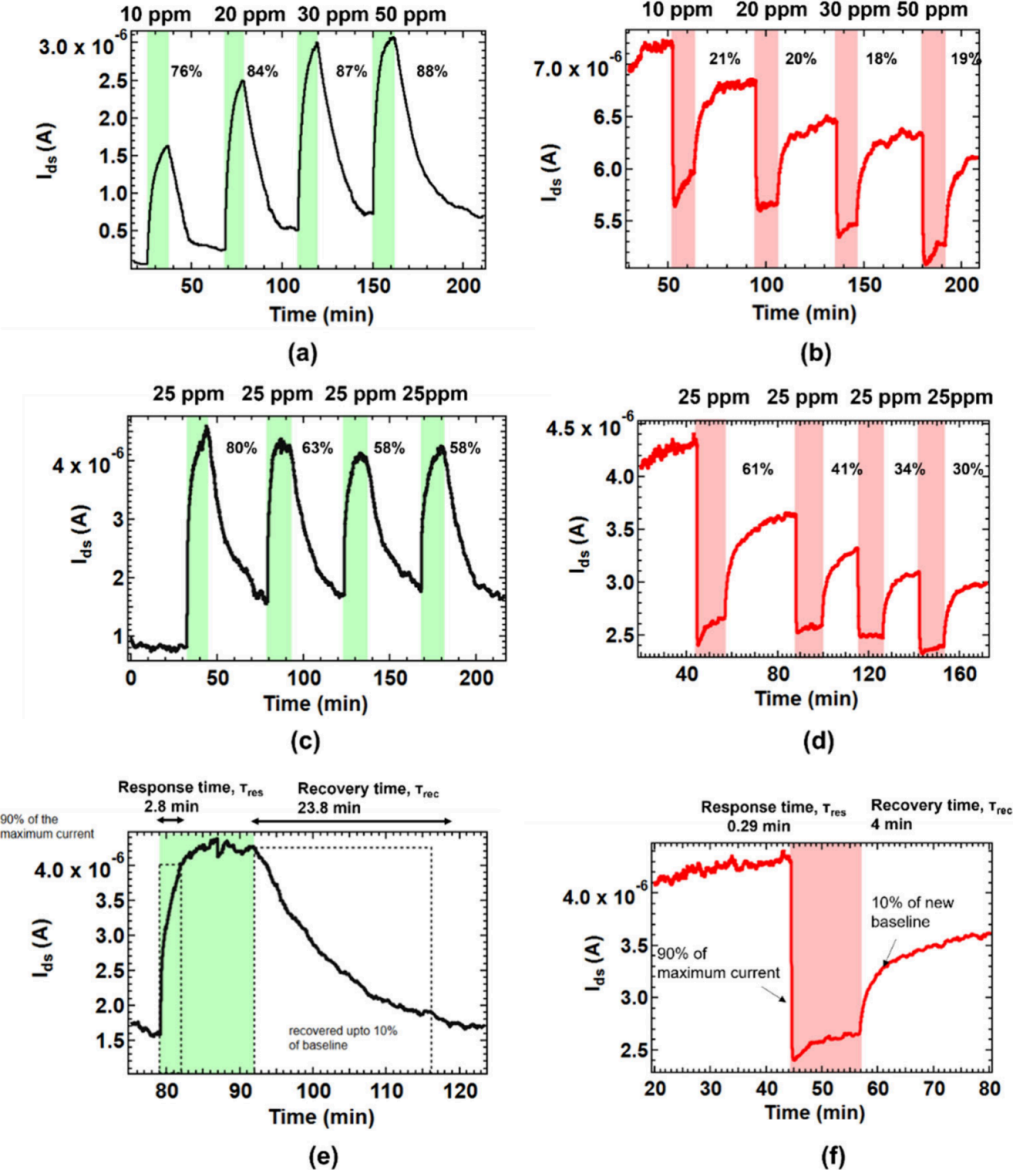
In the presence of UV light, current variation in response to 10 ppm, 20, 30, and 50 ppm of NH_3_ exposure for the (a) *n*-type conduction at *V*_ds_ = 1 V and *V*_gs_ = 40 V with exposure periods highlighted in green and (b) *p*-type conduction at *V*_ds_= 1 V and *V*_gs_ = −40 V with exposure periods highlighted in light red. Time-dependent current evolution during repetitive exposure to 25 ppm of NH_3_ for the (c) *n*-type conduction at *V*_ds_ = 1 V and *V*_gs_ = 40 V and (d) *p*-type conduction at *V*_ds_ = 1 V and *V*_gs_ = −40 V. (e) Response and recovery time for *n*-type conduction at *V*_ds_ = 1 V and *V*_gs_ = −40 V upon exposure to 25 ppm of NH_3_. (f) Response and recovery time for *p*-type conduction at *V*_ds_ = 1 V and *V*_gs_ = 40 V upon exposure to 25 ppm of NH_3_.

In the *n*-channel, a substantial variation in current was observed across different NH_3_ concentrations, with calculated responsivities ranging from 76 to 88% for NH_3_ levels between 10 and 50 ppm (Figure 4(a)). Under UV light, the *n*-channel demonstrated an almost 10-fold increase in response compared to its behavior in the absence of light. In contrast, the *p*-channel was less responsive, showing 18 to 21% across the same NH_3_ concentration range (Figure 4(b)). This asymmetry in response contrasts with the more balanced modulation observed in both channels when UV light was not applied. Notably, the sensor’s transient response did not increase linearly as NH_3_ concentrations rose in the high-concentration range.

To further investigate this effect, Si-JNTs were exposed to repeated pulses of 25 ppm of NH_3_ with 12 min durations (Figures 4(c) and (d)). As expected, the first NH_3_ exposure caused the strongest response, with 80% in the n-channel and 60% in the *p*-channel. However, subsequent exposure pulses diminished response signals, ranging from 63 to 58% in the *n*-channel and from 41 to 30% in the *p*-channel. This decline in response happens through two mechanisms, as supported by DFT calculations (see Figure 3 in the manuscript). First, NH_3_ molecules physiosorb onto the SiO_2_ surface, forming shallow trap states that enable reversible charge transfer through tunnelling. Second, NH_3_ chemically reacts with surface hydroxyl groups or defect sites to produce NH_4_^+^ species, leading to irreversible chemisorption and passivation of active sites. This process reduces the number of available sites for NH_3_ interaction over multiple cycles, lowering the responsivity. Additionally, UV exposure can cause permanent surface modifications, such as oxygen vacancies (E-centers) resulting from Si–O bond breaking in the oxide layer. These changes compromise the long-term stability of the material under UV-assisted conditions sensors.^46,62^ This accounts for the initially high responsiveness that gradually decreases with repeated exposures as sites become saturated or stabilized. A baseline drift in the current appears in the sensor response curves, likely caused by hysteresis in the transfer characteristics of the unpassivated Si-JNT, which affects both the *p*- and *n*-channels of the ambipolar transistor. The hysteresis change upon NH_3_ exposure (see Figure S2 in the Supporting Information) contributes to this baseline drift. Using passivated Si-JNTs with a thin, thermally grown oxide could provide a more stable response with minimal baseline shift. To address baseline drift, differential current can be employed as a sensing parameter, as it measures the rate of change rather than the absolute current (see Figure S6 in the Supporting Information for the variation in differential current over time under different NH_3_ exposures with UV light). This approach enhances robustness in dynamic sensing environments or those with fluctuating conditions.

The response and recovery times of the Si-JNT sensor were determined by analyzing the current variation upon exposure to 25 ppm of NH_3_ for both the *p*- and *n*-conduction channels of the Si-JNT device, as illustrated in Figures 4(e) and 4(f). The response and recovery times, defined as the durations required for 90% current variation at the rising or falling edges, were measured at 2.7 and 23.8 min, respectively, for the *n*-channel. In contrast, the *p*-channel exhibited significantly improved performance, with a response time of 0.26 min and a recovery time of 4 min. Notably, the ambipolar Si-JNT sensor displayed distinct characteristics in its *n*- and *p*-channel responses to NH_3_. The *n*-channel offers higher sensitivity, while the *p*-channel provides a faster response. This dual-responsive behavior gives the sensor an advantage over conventional uniresponse sensors by enabling the selection of optimal parameters from both channels. Specifically, the *n*-channel’s high responsivity and the *p*-channel’s rapid response time can be leveraged in the ambipolar Si-JNT, thereby enhancing the overall performance of the sensor platform.

The transfer characteristics of Si-JNT maintain their ambipolar nature when exposed to UV light and NH_3_. A similar hysteresis change is observed under UV illumination (λ = 254 nm) as in the non-UV scenario (see Figure S2 in Supporting Information). UV light photoassisted generation of electrons from E-centers in native SiO_2_, combined with altered adsorption–desorption dynamics of NH_3_, improves trap state passivation, resulting in a narrower hysteresis loop. The output characteristics of the ambipolar Si-JNT were also recorded under 254 nm UV light (see Figure S4­(b) in the Supporting Information). The device exhibits a clear complementary response: the drain current in hole conduction decreases while the current in the electron conduction channel increases as the NH_3_ concentration rises (0, 25, and 50 ppm). These current changes are more pronounced under UV light compared to dark conditions, across both conduction regimes. Additionally, we calculated the charge carrier numbers in the Si-JNT under UV light (see Table S3). Exposure to UV light amplifies ammonia’s modulation of charge carriers: the hole concentration drops to 0.8 of its initial value, while the electron concentration more than doubles at 50 ppm of NH_3_. This significant increase under UV suggests that the UV-activated surface (SiO_2_ with E-centers) boosts carrier generation and facilitates charge transfer via tunnelling between NH_3_ molecules and the silicon nanowire (supported by DFT calculations), indicating NH_3_ sensing through pseudomolecular doping in the Si nanowire channel.

Rapid detection of varying NH_3_ concentrations with high sensitivity, as well as continuous monitoring of concentration fluctuations, is crucial for implementing adequate safety measures and environmental surveillance. To achieve this, the response of ambipolar Si-JNTs to low NH_3_ concentrations (0.2 to 1.5 ppm) was investigated, as shown in Figure 5. Notably, both channels demonstrated sensitivity to NH_3_ concentrations as low as 0.2 ppm. Figure 5 presents the dynamic NH_3_ response of the Si-JNT sensor at ambient temperature, with current measurements taken under a constant source-to-drain voltage (*V*_ds_) of 1 V and source-to-gate voltage (*V*_gs_) of 40 V for *n*-conduction and −40 V for *p*-conduction. The sensing behavior exhibits repeatability for 0.8 ppm of NH_3_ (Figures 5(a) and 5(b)) in both conduction channels, indicating the sensor’s potential for repeated use without degradation in sensitivity. Upon exposure to 0.8 ppm of NH_3_, the device exhibited responsivities of 19.4 ± 1.5% for the hole channel and 16.5 ± 1.2% for the electron channel. Furthermore, the sensor’s response to different NH_3_ concentrations (0.2, 0.4, 0.8, and 1.5 ppm) is distinguishable in both conduction channels (Figures 5(c) and 5(d)), demonstrating its capability to detect even low concentrations of 0.2 ppm. The Si-JNT response and NH_3_ concentration correlation are plotted for both conduction channels in Figures 5(e) and 5(f). The observed linear relationship suggests that current variations are an effective parameter for NH_3_ detection within the low concentration range, reinforcing the sensor’s applicability in real-world monitoring scenarios.

**Figure 5:**
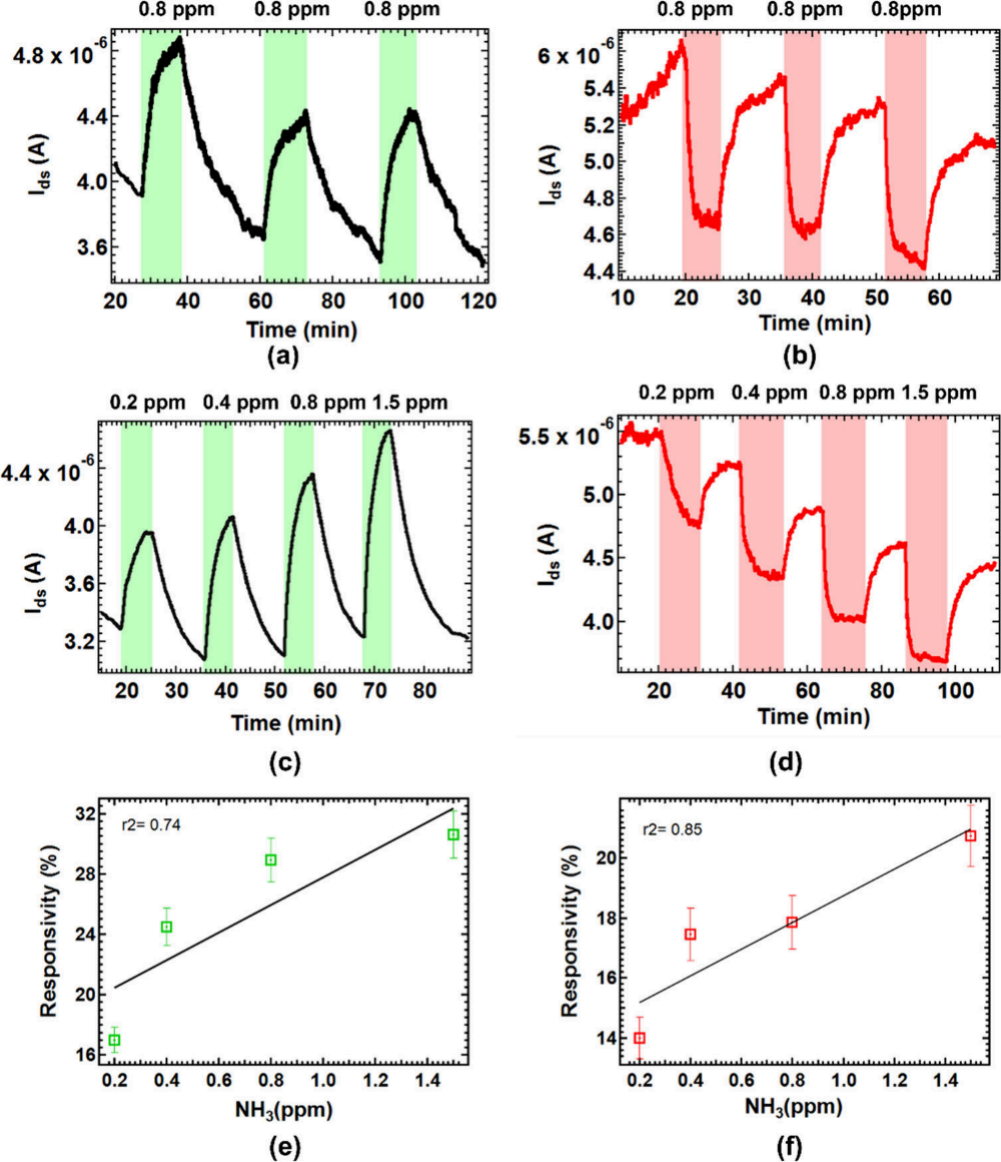
In the presence of UV light, time-dependent current response to repeated exposure of 0.8 ppm of NH_3_ in the (a) *n*-conduction channel at *V*_ds_ = 1 V and *V*_gs_ = 40 V, with exposure periods highlighted in green color and (b) *p*-conduction channel at *V*_ds_ = 1 V and *V*_gs_ = −40 V with exposure periods highlighted in light red color. (c) Current variation in the *n*-conduction channel upon exposure to NH_3_ concentrations of 0.2, 0.4, 0.8, and 1.5 ppm at *V*_ds_ = 1 V and *V*_gs_ = 40 V. (d) Current variation in the *p*-conduction channel upon exposure to NH_3_ concentrations of 0.2, 0.4, 0.8, and 1.5 ppm at *V*_ds_ = 1 V and *V*_gs_ = −40 V. (e) Responsivity of the *n*-conduction channel at different NH_3_ mixing ratios. (f) Responsivity of the *p*-conduction channel at different NH_3_ mixing ratios.

The Si-JNT sensor exhibits distinct sensing characteristics at low NH_3_ concentrations compared to higher concentrations of NH_3_. Unlike its behavior at high NH_3_ levels, the sensor demonstrates a consistent and linear response when exposed to low NH_3_ concentrations (0.2–1.5 ppm). This difference is likely due to the predominant role of physisorption (Path 1 in DFT) at lower concentrations. In contrast, at higher NH_3_ levels (10 to 50 ppm), physisorption and reactions with OH surface groups (Paths 1 and 2 in DFT) contribute to the sensing mechanism. The relative standard deviation (RSD) values remained consistently below 6%, demonstrating excellent repeatability. At 0.8 ppm of NH_3_, the *n*-channel had an RSD of 5.7%, whereas the *p*-channel showed a slightly lower variation of 3%, indicating greater stability. These findings confirm the sensor platform’s reliability and its potential for real-time, quantitative gas detection.

For low-concentration NH_3_ detection (0.8 ppm), the *n*-channel of the Si-JNT device exhibited a higher responsivity (19.4 ± 1.5%) compared to the *p*-channel (16.5 ± 1.2%). However, the *p*-channel demonstrated superior response and recovery times. Specifically, the *n*-channel had a response time of 3.0 min and a recovery time of 8.8 min, averaged for three subsequent 0.8 ppm of NH_3_ pulses. In contrast, the *p*-channel responded more rapidly, with a response time of 1.9 min and a recovery time of 2.9 min, averaged over three subsequent 0.8 ppm of NH_3_ concentrations. This trend aligns with the sensor’s behavior at higher NH_3_ concentrations, reinforcing the dual advantages of sensitivity and speed offered by the ambipolar Si-JNT sensor. The quicker response observed in the *p*-channel and the increased responsivity in the *n*-channel stem from fundamental differences in how NH_3_ influences charge carriers in ambipolar devices. During NH_3_ exposure, electrons are donated in the Si channel, likely through filled states within the SiO_2_ energy band gap (see Figure 3). This causes energy levels to shift, resulting in a higher influx of electron carriers and relatively fewer hole carriers (refer to Table S3 for carrier concentration calculations). As a result, the *n*-type operation exhibits more significant changes, producing a stronger sensor response. This behavior contrasts with our previous results involving oxidizing gases NO_2_,^49,50^ where a dominant change in Si-JNT was observed for *p*-type operation. The ambipolar Si-JNT transistor is mainly *n*-doped, with *p*-type conduction triggered by hole injection due to the low Schottky barrier height for holes during back-gate operation. In the *p*-conduction channel, holes are already minority carriers in *n*-doped Si. Therefore, electrons donated upon NH_3_ exposure can quickly neutralize injected holes, causing a rapid decrease in current and resulting in a swift response. We also calculated the theoretical limit of detection (LOD) using the standard deviation of the baseline.^63^ LOD is determined as 22 ppb for the *p*-channel and 33 ppb for the *n*-channel, underscoring the sensor’s effectiveness in measuring atmospheric NH_3_ levels. We compared the Si-JNT sensor’s performance with that of recent studies (see Tables S1 and S2 in the Supporting Information).

The mechanism behind the UV-enhanced response involves the creation of oxygen vacancies (E-centers) in the SiO_2_ layer.^64^ E-centers are identified as the most common natural defects in SiO_2_.^65^ UV exposure can generate defects on the native SiO_2_ surface and cause structural changes in amorphous SiO_2_, such as breaking Si–O bonds and forming reactive sites, which modify its properties.^66^ These changes largely explain the increased sensor signal observed. UV exposure induces oxygen vacancies, or E-centers, within SiO_2_. These vacancies create states in the oxide’s band gap that enable carrier tunnelling between the Si channel and adsorbed NH_3_ molecules (Path 1 in the DFT calculation, Figure 3). As a result, the Si-JNT sensor response is significantly enhanced. UV light (∼250 nm) provides enough energy to excite or ionize electrons at E-centers in the native oxide and generate electron–hole pairs in Si nanowires. The photoactivated electrons in E-centers can also take part in charge transfer reactions, in addition to those caused by NH_3_ adsorption. We observed a 2-fold increase in electron concentration upon exposure to NH_3_ under UV illumination, as indicated by output measurements (see Figure S4 in the Supporting Information). Refer to Table S3 in the Supporting Information for carrier concentrations under various testing conditions.

Moderate UV light at 254 nm is expected to partially penetrate the SiO_2_ native oxide layer. In ambipolar Si-JNTs without NH_3_, UV exposure consistently increases *p*-channel (hole) current while decreasing *n*-channel (electron) current, as shown in Figure S7 in the Supporting Information. This asymmetry indicates that UV light actively influences charge transport by altering band alignment or surface state populations. Since the native oxide on our Si nanowires is about 1–2 nm thick, it is plausible that 254 nm UV light can reach through this layer to interact with the surface and underlying Si. Similar phenomena have been observed in optoelectronic nanowire sensors, where native oxide-covered Si nanowires exhibit increased conductivity under UV light due to carrier generation and trap states modulation.^67^ Therefore, some UV-created holes in Si nanowires can recombine with electrons from the E-centers, while the other UV-induced electrons lead to a significant rise in n-channel conduction.

The formation of E-centers often coincides with the release of hydrogen atomic impurities in SiO_2_. Some hydrogen atoms can migrate to the oxide surface, helping form ammonium (NH_4_^+^) species, as shown in the DFT analysis (Path 2). To investigate the role of E-centers in UV-activated ammonia sensing, we tested Si-JNTs with about 10 nm of thermally grown SiO_2_ for NH_3_ sensing under UV light. Native oxide, formed at room temperature, is typically low density and structurally disordered, leading to many oxygen vacancies and dangling Si bonds (E′ centers). In contrast, oxide grown at high temperature (900 °C) can relax structurally and heal defects, creating a more stoichiometric, dense oxide with fewer defects E-centers.^68^ Due to the lower density of E-centers, Si-JNTs with thermally grown oxide are expected to be less sensitive to NH_3_. To verify this, we exposed Si-JNT devices with thermally grown oxide to 25 ppm of NH_3_ under UV light (see Figure S8 in the Supporting Information). As anticipated, native oxide Si-JNT devices exhibited significantly higher NH_3_ responsiveness under UV light (∼80%) compared to Si-JNTs with thermal oxide (∼10%). This indirectly indicates that UV-activatable E-centers in the SiO_2_ layer may play a role in NH_3_ sensing. Nonetheless, further spectroscopic studies of devices with different morphologies, before and after UV exposure, could better confirm the E-centers’ role in the sensing mechanism. These studies are planned for future research. Furthermore, UV irradiation has been reported to enhance the hydrophilicity of SiO_2_ surfaces, thereby improving surface stability and strengthening the physisorption of NH_3_ molecules. This effect aligns with experimental observations, demonstrating a higher sensor response and faster response times under UV illumination compared to measurements conducted without light. This behavior contrasts with the sensor’s response to the oxidizing gas NO_2_, where dominant charge carrier interactions differ.^49^

All sensor tests, including UV-assisted measurements, were conducted within three months of fabrication. The consistent responsiveness from various Si-JNT devices indicates low variability and stable performance. To further confirm device stability, additional comparative tests were performed for high and low NH_3_ concentrations. Si-JNT sensors maintained excellent stability after one month at 25 ppm of NH_3_, with negligible changes in responsivity (see Figure S9­(a) in Supporting Information). After 72 h, the response to 0.8 ppm of NH_3_ remained very similar to the initial, at 19.9 ±  6% and 23.7 ±  6%, respectively (see Figure S9­(b) in Supporting Information). Remarkably, even after 8 months, the Si-JNT showed a responsivity of 25  ±  6% to 0.8 ppm of NH_3_, comparable to the 19.4  ±  5.7% response of a freshly fabricated device under the same conditions. These results demonstrate the device’s ability to maintain sensing performance over time, despite exposure to handling and environmental conditions. Cycle stability was tested with three consecutive NH_3_ pulses at both high and low concentrations. As noted in the manuscript, a slight decrease in responsivity was observed at high concentrations (Figure 4). However, the response remained stable over three cycles at low concentrations. To evaluate repeatability, we calculated the RSD across multiple NH_3_ pulses at different concentrations for both *n*-type and *p*-type channels. The RSD values were consistently below 6%, indicating excellent reproducibility of the sensor signals.

### Selectivity of Si-JNT Sensors

The performance of the Si-JNT device was evaluated under both dry and humid air conditions, revealing a significant impact on its sensitivity to NH_3_ (see Figure S10 in Supporting Information). A comparison of the sensor’s response to 25 ppm of NH_3_ in dry air versus 50% relative humidity (RH) demonstrated a notable difference in responsivity. For example, the *n*-channel exhibited a strong response in dry air, with a relative responsivity of 82% upon NH_3_ exposure, whereas under 50% RH, the responsivity decreased to 48%. This matches common interpretations of competitive adsorption, where water molecules occupy active surface sites, blocking NH_3_ access or charge transfer, especially when surface sites are nearly saturated. Similar findings have been reported in hybrid polymer-based sensors, where humidity decreases NH_3_ sensitivity by disrupting surface binding interactions.^69^

At low NH_3_ levels (0.8 ppm), the effect reverses: responsivity rises from approximately 20% in dry air to about 40% at 50% RH (see Figure S10 in the Supporting Information). When concentrations are low, gas molecules seldom find active sites independently, and humidity can enhance the response by ionizing polar gases like NH_3_ (NH_3_ → NH_4_^+^). In humid conditions, an NH_3_ molecule may abstract a hydrogen atom from an OH group on the SiO_2_ surface, leading to the formation of an NH_4_^+^ species. This process has also been supported by DFT calculations as a potential pathway for ammonia detection. This behavior aligns with findings in polymer-based hybrid sensors and TiO_2_–PANI systems subjected to humidity conditions.^46^ To minimize humidity interference at high NH_3_ levels, we suggest two approaches: (1) applying hydrophobic surface passivation, such as fluorinated self-assembled monolayers, to repel water while allowing NH_3_ to reach reactive surfaces sites^70,71^ and (2) humidity-selective filtering layers, such as porous membranes or polymer coatings, which restrict moisture penetration while allowing analyte diffusion.^72^ These strategies can be applied in future sensor designs to improve performance in real-world settings. We also examined how Si-JNT sensors responded to a major pollutant, carbon monoxide (CO). Instead of showing a typical sensor response, the baseline steadily increased even after CO was removed (see Figure S11 in Supporting Information). This behavior could be due to CO interacting with Si dangling bonds and causing localized charge accumulation under UV light.

We evaluated the interaction of Si-JNTs with three other gases (NO_2_, CH_4_, and SO_2_) and NH_3_ to assess the sensor’s selectivity. A wide range of transistor parameters in the dual-responsive ambipolar Si-JNT were analyzed at a high concentration (25 ppm) to investigate potential interference from common atmospheric gases. Eight measurable and calculated responses were identified from the gas-Si-JNT interaction, with each gas producing a distinct response ″profile″ based on these parameters, as illustrated in Figure 6. The device’s sensitivity is determined by the lowest concentration that causes a measurable change in at least one parameter. Suppose the sensor responds exclusively to a single gas; a single parameter can be used for calibration. However, when multiple gases induce a response, full response profiles must be utilized to differentiate between analytes. Notably, Figure 6(a) shows that all sensor parameters react oppositely for NO_2_ compared to NH_3_. Also, NH_3_ can be distinguished from SO_2_ using five parameters (*g*_m_h_, *g*_m_e_, *I*_on_h_, μ_h_, and μ_e_) and CH_4_ using four (*g*_m_h_, *I*_on_h_, μ_h_, and *V*_th_e_), each showing opposite behavior. These variations enable the development of an algorithm to distinguish NH_3_ from other gases, provided the full range of mixing ratios and mixtures has been thoroughly tested to provide training data for a calibration model.

**Figure 6:**
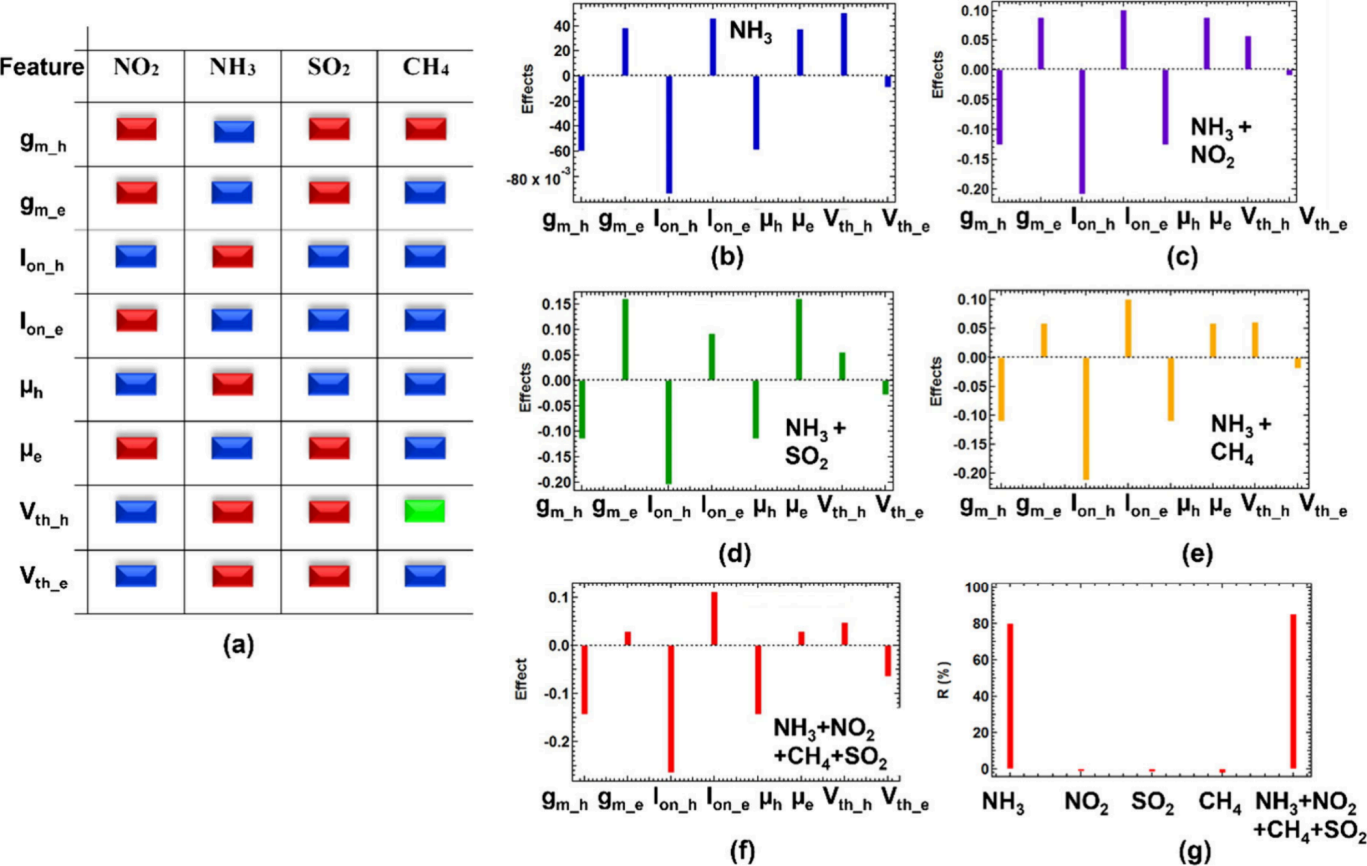
(a) Comparison of different Si-JNT parameters (on-current (*I*_on_), threshold voltage (*V*_th_) and mobility (μ)) upon exposure to NO_2_, NH_3_, SO_2_ and CH_4_ for both hole and electron conduction. The blue color indicates a positive effect; the red color indicates a negative effect, and green indicates a neutral effect. Relative effects of different Si-JNT parameters on (b) NH_3_ response, (c) NH_3_ + NO_2_, (d) NH_3_ + SO_2_ and (e) NH_3_ + CH_4_. (f) response profile for a mixture NH_3_ + CH_4_ + NO_2_ + SO_2_. (g) Comparison of responsivity for individual gases at 25 ppm and their different combinations at the same concentration in the presence of UV light.

A series of experiments was conducted using a design of experiments (DOE) approach to further analyze gas selectivity. A complete factorial design with four gases at two levels (0 and 25 ppm) resulted in 2^4^ = 16 tests, allowing for the evaluation of singular, binary, and tertiary gas interactions on sensor response. The experiments, conducted over 20 min with 5 min gas injection intervals, revealed interactive and nonadditive effects between gases, further highlighting the complex interplay in multigas environments. We have investigated the “effect” of individual gases and the mixture of gases on each ambipolar transistor parameter. In gas mixtures containing NH_3_ with NO_2_ (Figure 6(c)), SO_2_ (Figure 6(d)), and CH_4_ (Figure 6(e)), most sensor parameters exhibit a response similar to that of individual NH_3_ exposure (Figure 6(b)). However, other gases can either attenuate or amplify the response. For instance, NO_2_ reduces the impact on *V*_th_ in the hole-conduction channel compared to NH_3_ alone, whereas SO_2_ enhances mobility in the *n*-channel of the ambipolar transistor. Similarly, in the NH_3_–CH_4_ mixture, the response profile closely resembles NH_3_ alone (Figure 6(e)). In a four-gas mixture (Figure 6(f)), the NH_3_ dominates the overall profile, and the interference from the other gases is weak.

We investigated the transient sensor response of Si-JNTs to three potential interfering gases (NO_2_, CH_4_, and SO_2_) in addition to NH_3_, each at 25 ppm, through both individual and combined gas exposures, as shown in Figure S12 (see Supporting Information). For this analysis, we focused on the *n*-channel conduction of the Si-JNT. Significant responsivity was observed only for NH_3_, while the other gases produced negligible signal changes. Gas mixtures containing 25 ppm each of NO_2_, NH_3_, SO_2_, and CH_4_ and 25 ppm of NH_3_ alone exhibited a response nearly identical to that of NH_3_ alone (Figure 6(g)). Additionally, NO_2_, SO_2_, and CH_4_ individually showed very low responsivity. A comparative analysis (Figure 6(g)) showed that both NH_3_ and the gas mixture produced a similar responsivity of 80 ± 5% in the *n*-channel conduction of the Si-JNT across both high and low NH_3_ concentrations. This finding supports the suggestion that Si-JNT have the capability for selective NH_3_ detection, even in complex gas environments. In sensing applications, the presence of NH_3_ in a gas mixture is recognized by its distinctive response profile, although other gases can affect this profile. Nonetheless, no other gas is expected to produce identical changes across all measured parameters. This indicates that different electrical parameters respond uniquely to various gases, and the combined response forms a fingerprint that varies per gas or mixture. We demonstrate that certain parameters change monotonically with NH_3_ concentration, and response patterns differ among gases and mixtures. The sensor responses were analyzed in two ways: (i) by examining the overall response profile changes and (ii) by assessing how each response feature depends on different gas mixtures. In these experiments, each gas was introduced at either 0 (ZA) or 1 ppm, with various combinations, and the transfer characteristics of the Si-JNTs were recorded during exposure. The experimental setups, parameter changes, and responses are detailed in Tables S4 and S5 in the Supporting Information. This approach benefits from revealing interactions and nonadditive effects between gases by comparing high responses (+1, 1 ppm) with low responses (−1, ZA) across all parameters. The response variables include both measured and derived parameters from the transfer curve. While our results suggest that selective NH_3_ detection is feasible, complete quantification in unknown mixtures will require further experiments with varying concentrations of interfering gases to create reliable calibration models, a future task. Currently, our findings demonstrate the potential to identify parameter response profiles for gas mixtures, establishing a foundation for algorithms such as decision trees to detect and quantify NH_3_ in complex environments.

## Conclusion

The paper highlights the potential of molecular doping to modulate the properties of ambipolar Si-JNTs in both electron and hole conduction modes through interaction with NH_3_. NH_3_ exposure caused dynamic changes in key transistor parameters (*I*_on_, *V*_th_, and μ) across the*p*- and *n*-transport channels of the ambipolar Si-JNT. Specifically, NH_3_ caused a decrease in *I*_on_ in the negative *V*_*gs*_ region (*p*-channel) while increasing *I*_on_ in the positive *V*_gs_ region (*n*-channel). Utilizing NH_3_-induced molecular doping, we demonstrated the capability of ambipolar Si-JNTs as dual-response sensors for detecting NH_3_, a critical air pollutant. These sensors exhibited high sensitivity and responsivity, as well as fast recovery, at concentrations ranging from 10 to 50 ppm. Under UV illumination, their detection range extended from 0.2 to 50 ppm in both conduction channels. The hole conduction channel displayed rapid response times (1.91 min for 0.8 ppm of NH_3_ and 0.29 min for 25 ppm of NH_3_), while the electron conduction channel exhibited high responsivity (80% for 25 ppm of NH_3_ and 25% for 0.8 ppm of NH_3_). The *p*-channel demonstrated a significantly faster recovery (2.96 min) than the *n*-channel (8.79 min) upon 0.8 ppm of NH_3_ exposure. We further analyzed the sensor’s response in diverse gas environments, distinguishing NH_3_ signals from interfering gases by leveraging the extensive parameter space of the ambipolar Si-JNT. These measured parameters provide the foundation for a multivariate calibration model, enhancing NH_3_ detection selectivity and sensitivity. Given its ability to detect a wide range of NH_3_ concentrations with fast response and recovery times, the Si-JNT sensor is a promising candidate for air quality monitoring and field measurements. Future research will focus on functionalizing Si-JNTs with organic coatings to refine gas-surface interactions, improving NH_3_ detection limits and selectivity in complex environmental conditions.

## Supplementary Material



## Data Availability

The datasets generated during and/or analyzed during the current study are available in the RADICAL Zenodo repository, https://zenodo.org/communities/radical/.

## Notes

Comprehensive literature review table on various NH_3_ sensors, the output characteristics of Si-JNT devices, transfer characteristics illustrating hysteresis, sensor stability measurements, tests involving Si-JNT with thermally grown oxide, as well as the effects of humidity and CO. It also includes a comparison of Si-JNT responses to different gases and a detailed chart on selectivity tests. (PDF)

## ¶

We dedicate this work to the memory of Professor Yordan M. Georgiev, whose expertise in nanofabrication, thoughtful mentorship, and scientific vision were central to this research. Yordan was not only a valued collaborator but also a great friend and colleague to us all. His passing is a profound loss to our team and the broader scientific community. We are deeply grateful for the time we shared with him and remain inspired by his lasting contributions to the field.
