# Supplementary material for: Ammonia Sensing via Pseudo Molecular Doping in UV-Activated Ambipolar Silicon Nanowire Transistors
Source: ACS Appl Mater Interfaces. 2025 Jul 24;17(31):44686–98. doi: 10.1021/acsami.5c08140 (PMC12332839; doi:10.1021/acsami.5c08140)
Supplement: Supplementary file 1 [file am5c08140_si_001.pdf]

Supporting Information

Ammonia Sensing via Pseudo Molecular Doping in UV-Activated Ambipolar Silicon Nanowire Transistors

Vaishali Vardhan [1,2], Subhajit Biswas[1,2], Leonidas Tsetseris[3], Sayantan Ghosh[4,5], Ahmad Echresh[4], S. Hellebust[1,2], Rene Huebner[4], Yordan M. Georgiev[4,6,†] and Justin D. Holmes[1,2*]*

[1]School of Chemistry, University College Cork, Cork, T12 YN60, Ireland.

[2]Environmental Research Institute, University College Cork, Cork, T23 XE10, Ireland.

[3]Department of Physics, School of Applied Mathematical and Physical Sciences, National Technical University of Athens, Athens 15780, Greece.

[4]Institute of Ion Beam Physics and Materials Research, Helmholtz-Zentrum Dresden Rossendorf, 01328, Dresden, Germany.

[5]Technische Universität Dresden, Dresden, 01069, Germany

[6]Institute of Electronics at the Bulgarian Academy of Sciences, 1784 Sofia, Bulgaria

† Deceased. This work is dedicated to the memory of Professor Yordan M. Georgiev.

*Correspondence: s.biswas@ucc.ie or j.holmes@ucc.ie

Figures

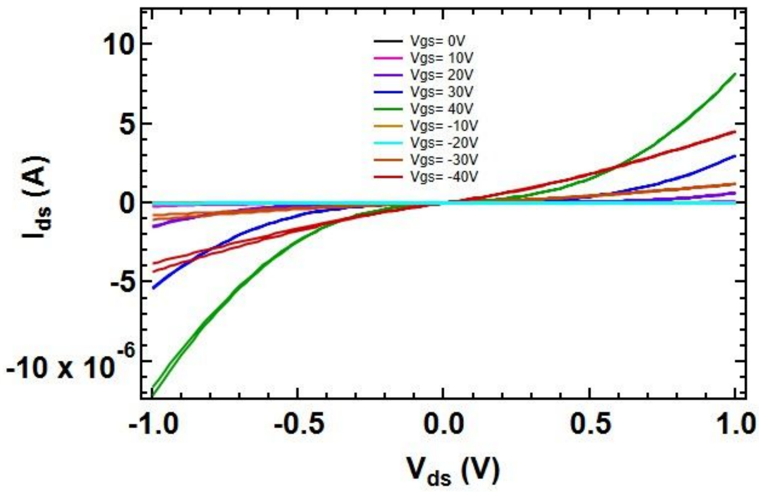

Figure S1. Output Characteristics of a Si-JNT device with drain voltage (V_{ds}) swept from -1 to 1 V at varying gate voltages (V_{gs}).

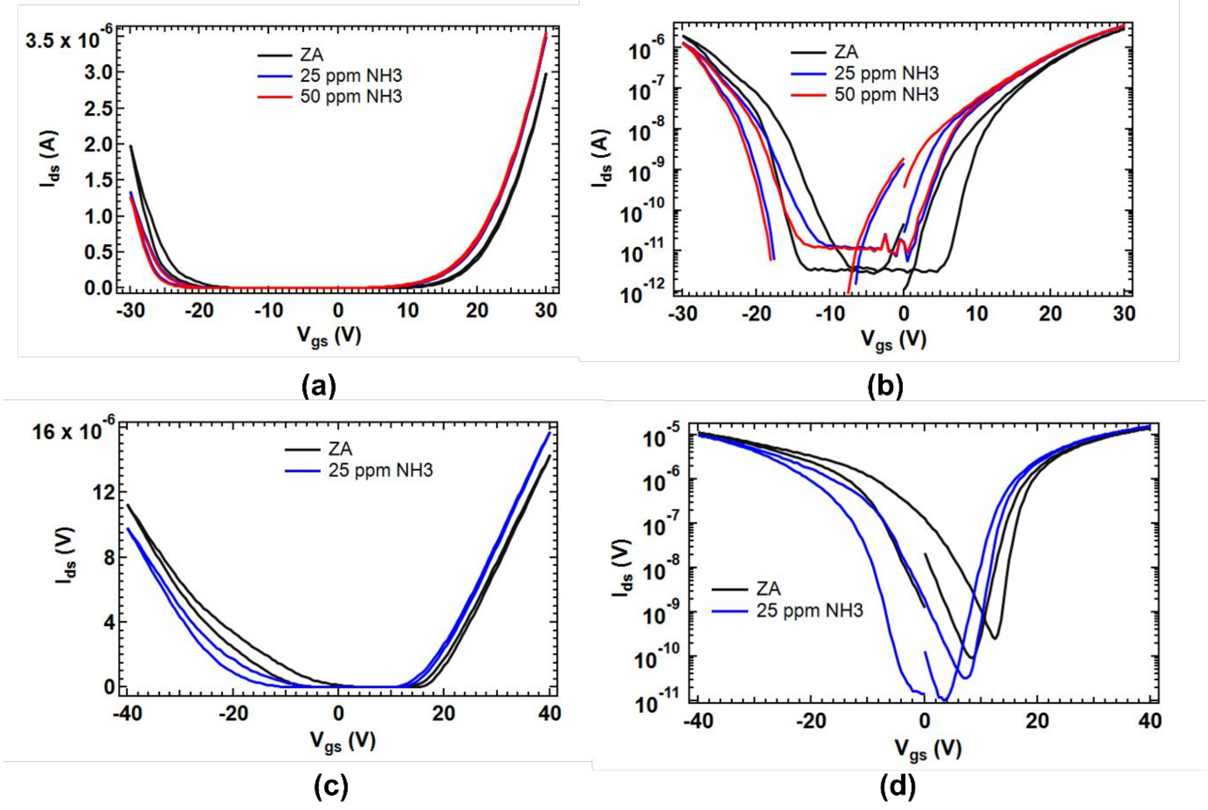

Figure S2. Change in *I-V* characteristics and hysteresis upon NH_3 exposure for two different devices: (a) linear scale and (b) log scale for ZA, 25 ppm, and 50 ppm NH_3 (Device 1); (c) linear scale and (d) log scale for ZA and 25 ppm NH_3 (Device 2).

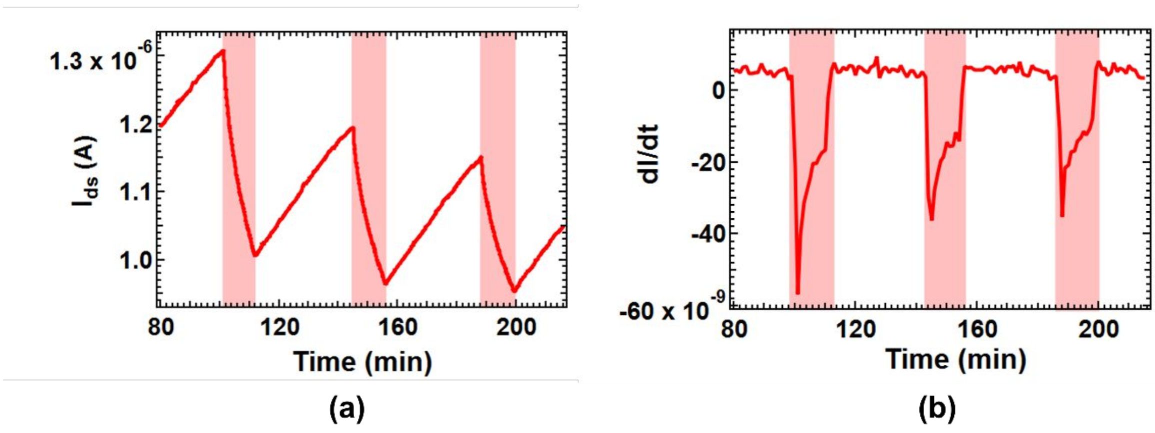

Figure S3. (a) Repeatability test for NH_3 exposure at 25 ppm (indicated by pink bars) for 10 min on the *p*-channel of the Si-JNT at V_{gs} = -40 V. (b) Differential current for 25 ppm of NH_3 exposure on the *p*-channel of Si-JNT at V_{gs} = -40 V.

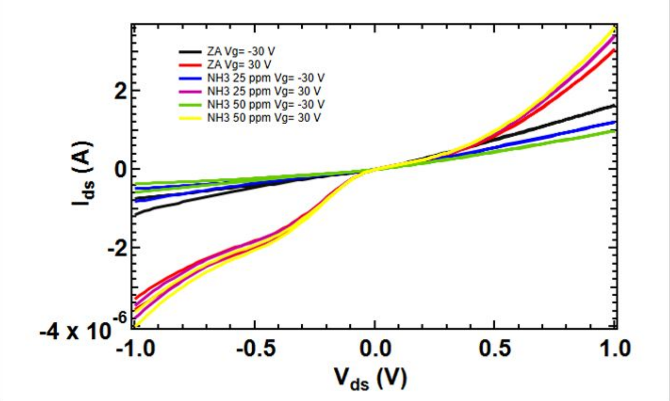

(a)

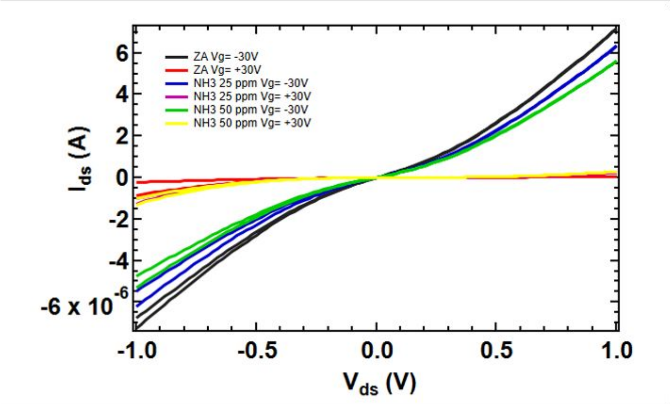

(b)

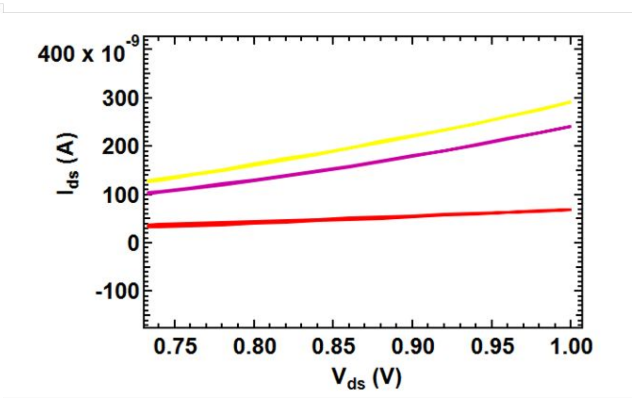

(c)

Figure S4. (a) Output characteristics of a Si-JNT device with drain voltage (V_{ds}) swept from -1 to 1 V at various gate voltages (V_{gs}) for ZA and different NH_3 concentrations under dark conditions. (b) Output characteristics of a Si-JNT device with drain voltage (V_{ds}) swept from -1 to 1 V at various gate voltages (V_{gs}) for ZA and different NH_3 concentrations under UV light (254 nm), and (c) a zoomed view of (b) from 0.75 to 1 V to observe changes in current.

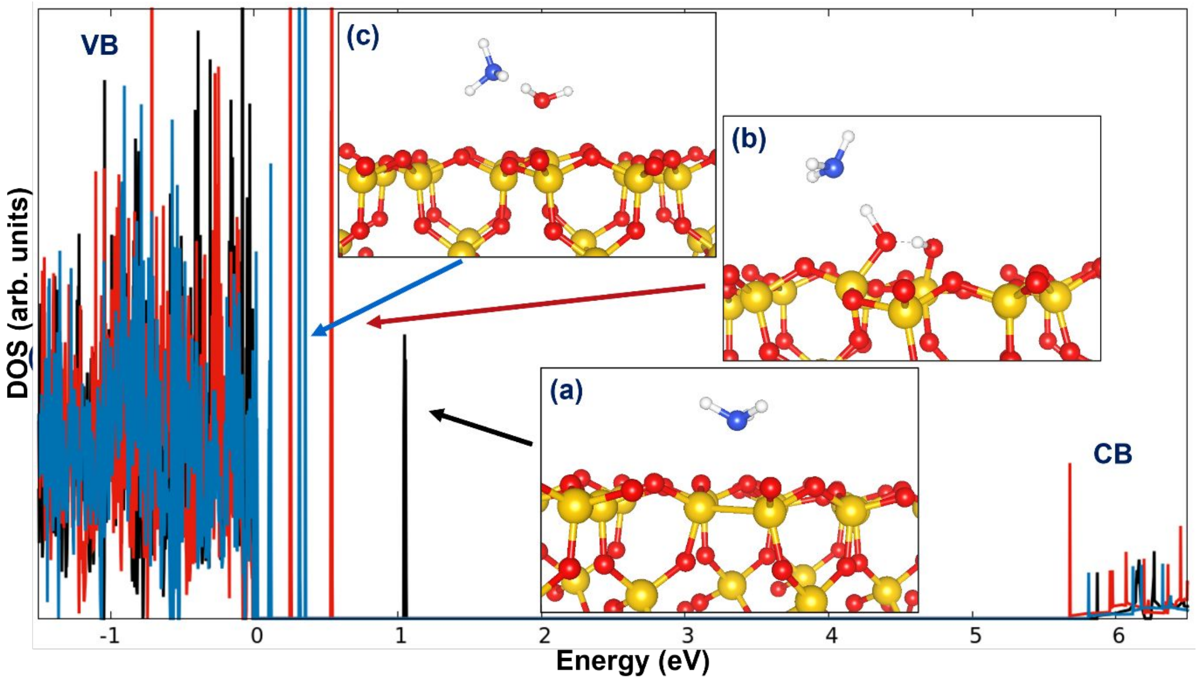

Figure S5. Electronic densities of states (DOS, in arbitrary units) for the NH_3 physisorbed configurations shown in insets: (a) an NH_3 molecule over a surface oxygen vacancy [red line], (b) an NH_3 molecule over a pair of surface hydroxyl groups [blue line], (c) an NH_3 molecule next to an adsorbed H_2O molecule (Si: yellow, O: red, N: blue, H: white spheres) . The zero of energy is set at the valence band maximum of SiO_2 (VB and CB are, respectively, the valence band and the conduction band). The arrows show filled NH_3-related states within the SiO_2 band gap.

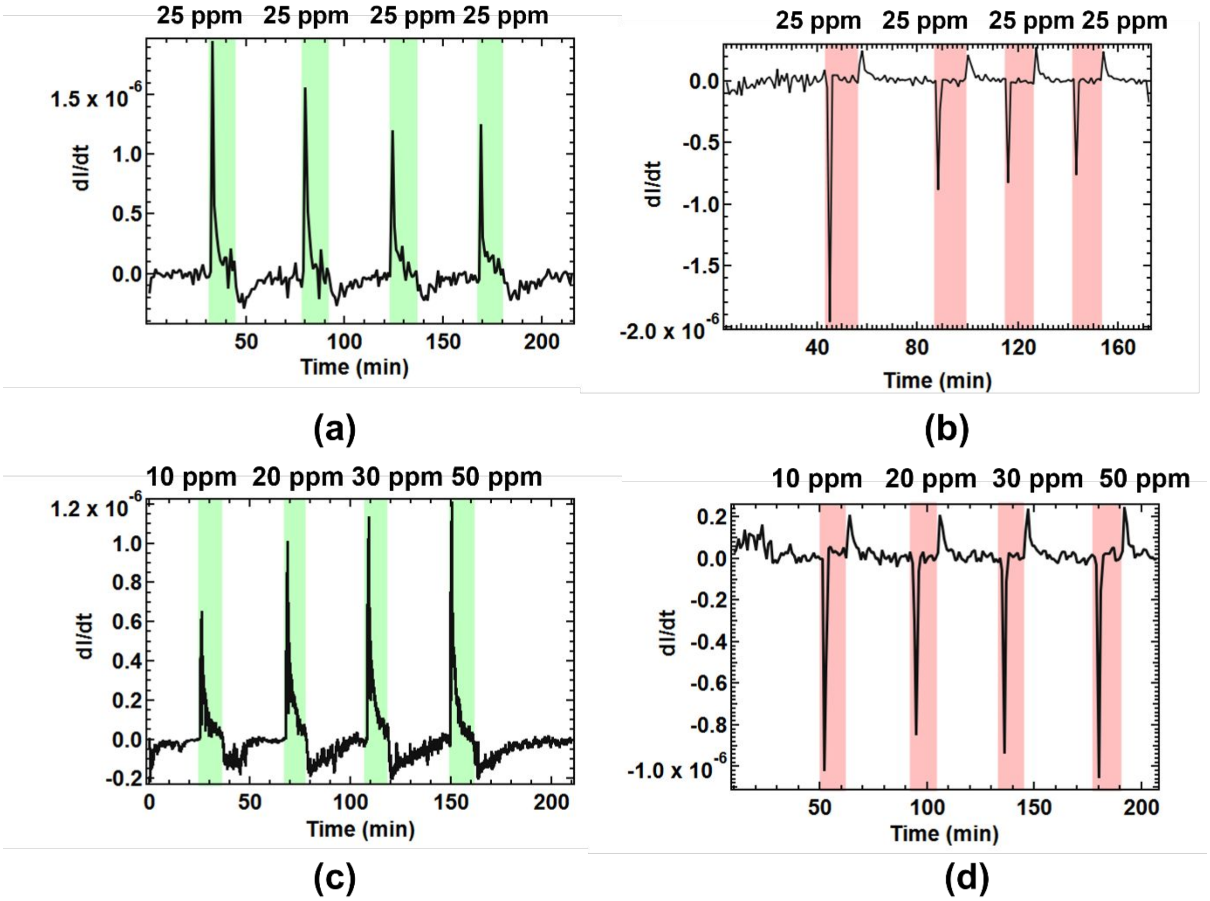

Figure S6. Differential current response under UV illumination to 25 ppm NH_3 for (a) *n*-type and (b) *p*-type conduction. Differential current response under UV illumination to varying NH_3 concentrations (10, 20, 30, and 50 ppm) for (c) *n*-type and (d) *p*-type conduction.

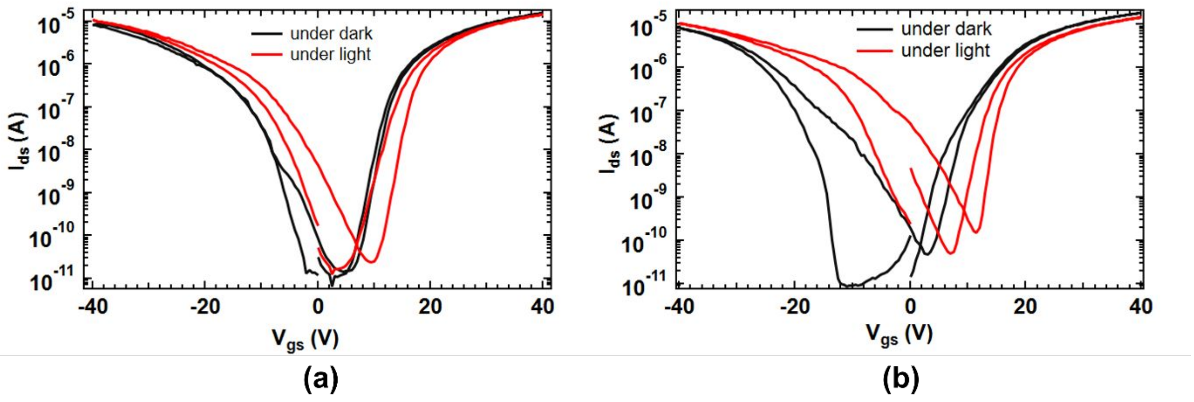

Figure S7. Evolution of *I-V* characteristics for two different native oxide Si-JNT devices measured under dark and UV-illuminated conditions: (a) Device 1 and (b) Device 2.

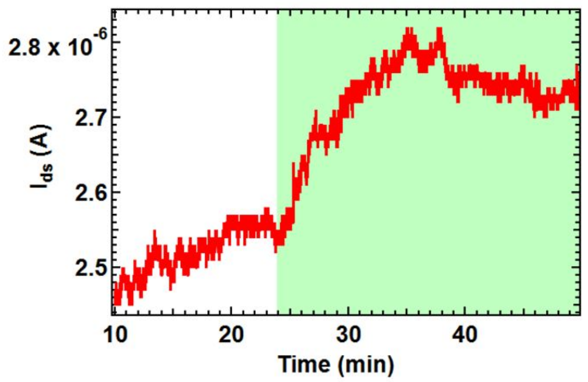

(a)

NO Si JNT	p-type	n-type
Under dark	22%	
Under light	42%	82%
10 nm Si JNT		
Under dark		~1.5%
Under light		9.5%

(b)

Figure S8. (a) Time versus current plot for Si-JNT with 10 nm thermal oxide during 25 ppm NH_3 exposure at V_{gs} = 40 V and V_{ds} = 1 V for the *n*-channel. The table in part (b) summarises the sensing performance of the native oxide and thermal oxide devices.

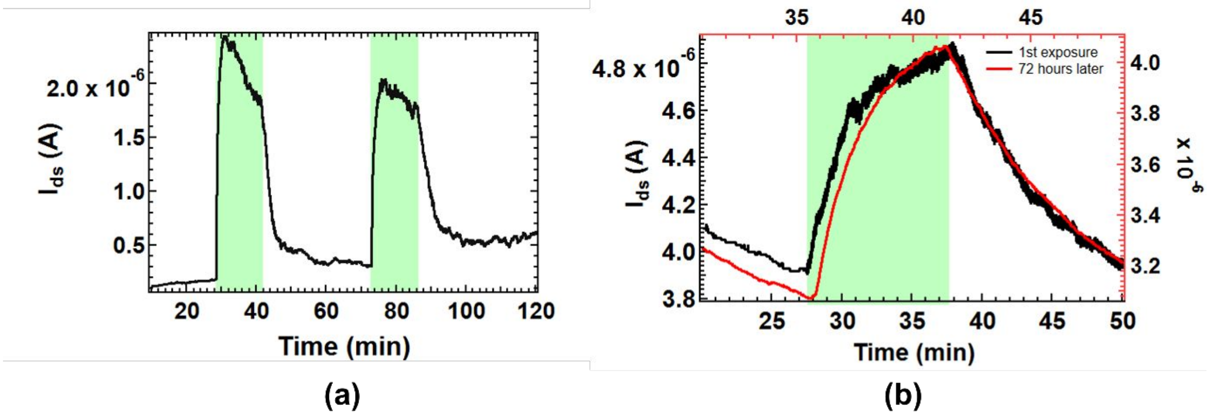

Figure S9. (a) Exposure to 25 ppm of NH_3 after one month (of initial NH_3 exposure) at V_{ds} = 1 V and V_{gs} = 40 V for the *n*-channel. (b) Stability test for 0.8 ppm NH_3 exposure on the first day and after 72 h at V_{gs} = 40 V and I_{ds} = 1 V for the *n*-channel.

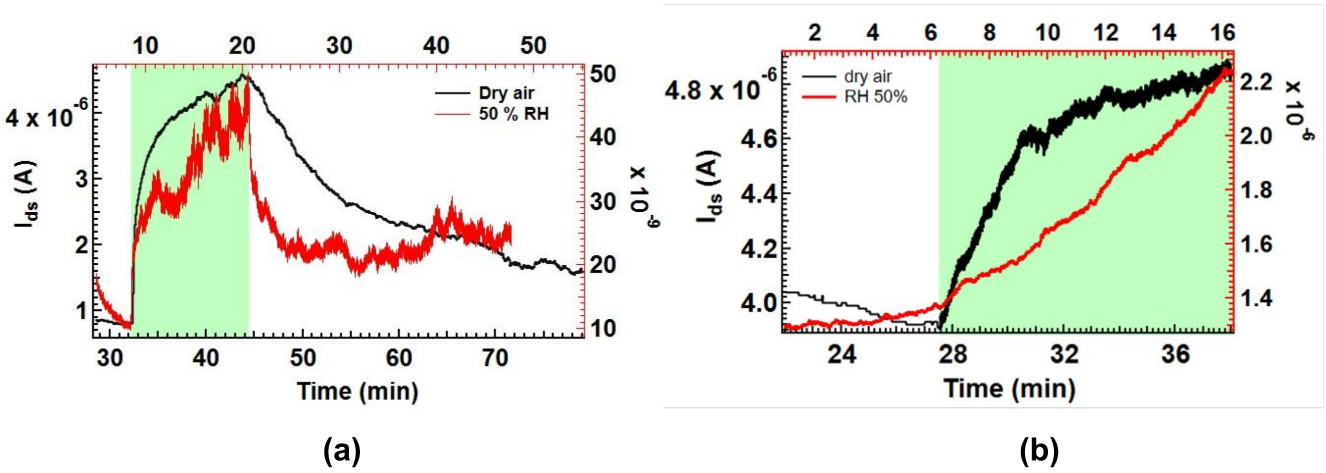

Figure S10. Comparison of current and responsivity during (a) 25 ppm and (b) 0.8 ppm NH_3 exposure under dry conditions (black line) and 50 % relative humidity (red line) for *n*-type conduction at V_{ds} = 1 V and V_{gs} = 40 V, with gas exposure highlighted in green in the presence of UV light.

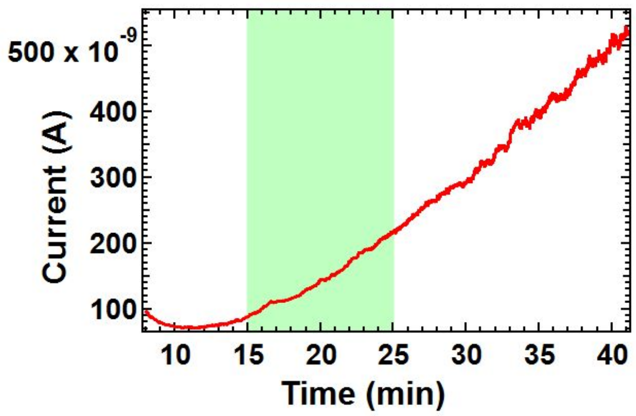

Figure S11. Response of Si-JNT sensor (*n*-channel) to 0.8 ppm CO at V_{ds} = 1 V and V_{gs} = 40 V.

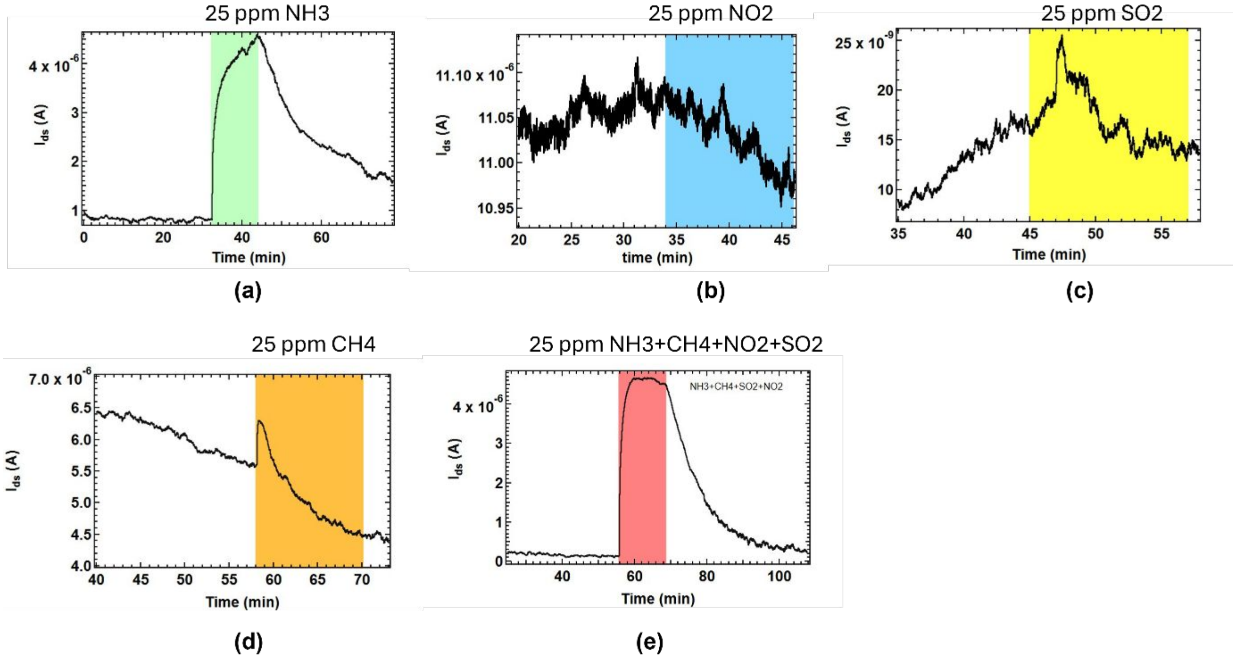

Figure S12. Response of Si-JNT devices to 25 ppm of various gases under *n*-type conduction at V_{ds} = 1 V and V_{gs} = 40 V: (a) NH_3, (b) NO_2, (c) SO_2, (d) CH_4, and (e) their mixtures. Gas exposure periods are indicated by coloured highlight bars.

Tables

Table S1. A Comparison of Si Platforms for NH_3 Sensing.

Device	Type	Surface functionalisation	Fabrication method	Operational temperature	Response time	Recovery time	Sensitivity	Range	Stability	Responsivity (%)	Reference
Ge-NP SiNW	FET	Ge NP	metal-assisted chemical etching (MACE)	RT	70 s for SiNW<br>220 s (Ge-SiNW	~400 s for 1 ppm NH_3	1 ppm	1-11ppm			(Sultan *et al.* 2025)
RGO/ZnO @SiNWs	Chemiresistor	RGO/ZnO	MACE	RT	3s for 0.01 ppm, 6s for 5 ppm	6 s for 0.01 ppm, 12 s for 5 ppm	0.01 ppm	0.01-5 ppm	10 days	21% at 0.01 ppm, 176 % at 5 ppm	(Yogi *et al.* 2024)
SWCNT-COOH	Si pillars, Si nanotubes	COOH	photolithography	RT	~200 s for 10 ppm	~200 s for 10 ppm	10 ppm	10-75 ppm (at 60%rh)		27~% Planar and 32% for Pillar at 75 ppm	(Kim H. *et al.*, 2024)
(SINWs)-(PANI)	Nanowire-polymer heterostructure	Polyaniline	Heterostructure fabrication	RT	~93 s for 10 ppm	~515 s for 10 ppm	10 ppm	10-100 ppm		150% for 100 ppm NH3	(Nath and Sarkar, 2023)
SiNW-PPy	dual-MACE for SiNW	Polypyrrole		RT	<30 s for 1 ppm NH3	~220s for 1 ppm NH3	130 ppb	1ppm-10ppm			(Qin *et al.*, 2018)
Self-aligned SiNW	FET device	-	IPSLS growth	RT	6 min (for 100 ppm NH3)	24 min (for 100 ppm NH3)	100 ppbv	0.1-100 ppm	180 days	7 % at 0.1 ppm NH_3<br>75.8% at 100 ppm NH_3	(Song *et al.*, 2021a)
AuNP-decorated Si-NWs	FET	-	EBL etching	RT			1 ppm	1-50 ppm	120 days	65 % at 1ppm 3500% at 50ppm	(Kim *et al.*, 2020)
Vertical Si-NW	resistance	-	MACE-itching	RT	9 s for 1ppm	150 s for 1 ppm	1 ppm	0.3 ppm-10ppm	22 days	1.2% at 1 ppm	(Qin *et al.*, 2017)
		Ag			<1 s for 0.3 ppm	5 s for 1 ppm	0.3 ppm				
Vertical porous Si-NW	resistance		Solution itching	RT	<25s for 50ppm NH3	10s for 50 ppm NH3	5 ppm	5-100 ppm	180 days	1.7 % for 50 ppm nh3	(Zhu, Liu, *et al.*, 2017)
Vertical Si-NWs	resistance	n-WO3	electrochemical etching	100 C	85s	2700 s	100 ppm	100 ppm	40 days	19.9	(Mhamdi *et al.*, no date)
random planar SiNWs	resistance		VLS growth	RT	400 s For 175 ppm NH3	600s For 175 ppm NH3	175 ppm	175-700 ppm		2.5	(Ni *et al.*, 2012b)
random planar Te-decorated SiNWs	resistance		Oxide assisted growth	RT	5s	8s	10 ppm	10-400ppm		30 % for 10 ppm NH3	(Yang *et al.*, 2013)
self-aligned SiNWs	resistance		EBL etching	RT	~500s (plane device) ~50s (75 nm nw)	~500s(plane device) ~300s(75 nm nw)	250 ppm	250 ppm		1.2% (plane device) 19.7% (with 75 nm nw)	(Wan *et al.*, 2009)

Table S2. A Comparison of Si Platforms for NH_3 Sensing in the presence of UV light.

Device	Type	Surface functionalization	Fabrication method	Operational temperature	Response time	Recovery time	Sensitivity	Range	Stability	Responsivity (%)	Reference
Se-hyperdoped silicon	Conductive gas sensor	none	Pulsed laser ablation (Se-hyperdoping)	RT	~10 seconds for > 200 ppm NH_3 =		100 ppm	10–500 ppm	1 month (degradation after 1 month)	2.6% for 50 ppm NH_3	(Liu *et al.*, 2018)
Sulphur-hyperdoped Si	conductometric sensor	none			2.3 min For 50 ppm NH_3	20.7 min for 50 ppm of NH_3	1 ppm	1ppm-50ppm		~75 % for 50ppm NH_3	(Liu *et al.*, 2019)
TiO_2/Ti_3C_2Tx MXene heterostructure	Resistive gas sensor		UV illumination-enhanced	RT	10 seconds (for 30 ppm NH_3)	5 seconds (for 30 ppm NH_3)	50 ppb	50 ppb – 30 ppm			(Zhang *et al.*, 2022)
TiO_2/PANI core-shell nanofibers	Chemiresistive gas sensor		Electrospinning for TiO_2, in-situ polymerization of PANI	RT	68–48 seconds (under UV, for 50 ppb to 40 ppm NH_3)	74–95 seconds (without UV), 28–51 seconds (under UV)	50 ppb	50 ppb – 40 ppm	Stable for 2 months (14–18% degradation over time)		(Seif, Nikfarjam and ghassem, 2019)
Si-NW	FET	none	UV-lithography	RT	1.91 min p-side and 2.96 min n-side For 0.8 ppm NH_3 under UV	2.96 min (p-side) and 8.79 min for 0.8 ppm NH_3 under UV	200 ppb	200 ppb-50 ppm	180 days	80-92% (25 ppm NH_3) 20-30% for 0.8 ppm NH_3	This work

Table S3. Change in carrier concentration under UV conditions calculated from the output characteristics of Si-JNTs.

	N_h (cm^{-3})	N_e (cm^{-3})
**ZA**	1.95×10^{19}	3.99×10^{18}
**NH_3 25 ppm**	1.72×10^{19}	6.40×10^{18}
**NH_3 50 ppm**	1.55×10^{19}	8.10×10^{18}

N_h: hole concentration (cm^{-3}); N_e: electron concentration (cm^{-3})

Table S4. Summary of changes in various parameters following exposure to different gases and gas mixtures. "-1" indicates 0 ppm addition, while "1" indicates 1 ppm addition.

Exp. No	NO2	NH3	SO2	CH4	Ion_left	Ion_right	mu_left	mu_right	Vtleft	Vtright
1	-1	-1	-1	-1	0	0	0	0	0	0
2	1	-1	-1	-1	0.250438	-0.04237	0.171597	-0.01186	-0.01449	0.013481
3	-1	1	-1	-1	-0.09386	0.045872	-0.05852	0.03736	0.050619	-0.00887
4	1	1	-1	-1	0.021277	0.030675	0.019837	-0.00978	0.011434	-0.06188
5	-1	-1	1	-1	0.036036	0.05168	0.038649	0.063227	0.002796	0.015122
6	1	-1	1	-1	-0.20784	0.100509	-0.12486	0.088464	0.057558	-0.0081
7	-1	1	1	-1	0.022631	0.003569	0.03684	-0.00128	0.004759	-0.0051
8	1	1	1	-1	0.049541	-0.02999	0.023588	-0.02165	-0.01904	0.012395
9	-1	-1	-1	1	-0.204	0.092581	-0.11344	0.1611	0.05526	-0.02739
10	1	-1	-1	1	-0.21177	0.100661	-0.11	0.058645	0.061251	-0.01784
11	-1	1	-1	1	0.058295	-0.02033	0.048766	0.004023	-0.00567	0.017233
12	1	1	-1	1	0.188144	0.083563	0.203591	0.246512	0.015577	0.032456
13	-1	-1	1	1	-0.02919	0.013702	0.064065	0.009924	0.027967	-0.00556
14	1	-1	1	1	0.161721	0.115438	0.163094	0.413981	0.009773	0.059173
15	-1	1	1	1	0.01146	-0.00706	-0.05931	-0.00065	-0.03527	0.009399
16	1	1	1	1	-0.26474	0.111303	-0.14315	0.028631	0.046709	-0.06364

Table S5. Sensor tests are conducted using a full factorial design involving four gases for Si-JNT devices with native oxide and thermally grown oxide, varied at two levels: -1 (0 ppm) and +1 (1 ppm). Interactive effects between 2, 3, and 4 gases are included in columns 5-16.

	NO2 (1)	NH3 (2)	SO2 (3)	CH4 (4)	1x2	1x3	1x4	2x3	2x4	3x4	1x2x3	2x3x4	1x2x4	2x3x4	1x2x3x4
**1**	-1	-1	-1	-1	1	1	1	1	1	1	-1	-1	-1	-1	1
**2**	1	-1	-1	-1	-1	-1	-1	1	1	1	1	-1	1	-1	-1
**3**	-1	1	-1	-1	-1	1	1	-1	-1	1	-1	1	1	1	-1
**4**	1	1	-1	-1	1	-1	-1	-1	-1	1	-1	1	-1	1	1
**5**	-1	-1	1	-1	1	-1	1	-1	1	-1	1	1	-1	1	1
**6**	1	-1	1	-1	-1	1	-1	-1	1	-1	-1	1	1	1	1
**7**	-1	1	1	-1	-1	-1	1	1	-1	-1	-1	-1	1	-1	1
**8**	1	1	1	-1	1	1	-1	1	-1	-1	1	-1	-1	-1	-1
**9**	-1	-1	-1	1	1	1	-1	1	-1	-1	-1	1	1	1	-1
**10**	1	-1	-1	1	-1	-1	1	1	-1	-1	1	1	-1	1	1
**11**	-1	1	-1	1	-1	1	-1	-1	-1	-1	1	-1	-1	-1	1
**12**	1	1	-1	1	1	-1	1	-1	1	-1	-1	-1	1	-1	-1
**13**	-1	-1	1	1	1	-1	-1	-1	-1	1	1	-1	1	-1	1
**14**	1	-1	1	1	-1	1	1	-1	-1	1	-1	-1	-1	-1	-1
**15**	-1	1	1	1	-1	-1	-1	1	1	1	-1	1	-1	1	-1
**16**	1	1	1	1	1	1	1	1	1	1	1	1	1	1	1
